# Four genes encoding MYB28, a major transcriptional regulator of the aliphatic glucosinolate pathway, are differentially expressed in the allopolyploid *Brassica juncea*

**DOI:** 10.1093/jxb/ert280

**Published:** 2013-09-16

**Authors:** Rehna Augustine, Manoj Majee, Jonathan Gershenzon, Naveen C. Bisht

**Affiliations:** ^1^National Institute of Plant Genome Research, Aruna Asaf Ali Marg, New Delhi 110067, India; ^2^Department of Biochemistry, Max Planck Institute for Chemical Ecology, Hans-Knöll-Straße 8, Jena, Germany

**Keywords:** *Brassica juncea*, expression partitioning, glucosinolates, *MYB28*, transcription factor.

## Abstract

Glucosinolates are Capparales-specific secondary metabolites that have immense potential in human health and agriculture. Unlike *Arabidopsis thaliana*, our knowledge about glucosinolate regulators in the *Brassica* crops is sparse. In the current study, four *MYB28* homologues were identified (*BjuMYB28-1,-2,-3,-4*) from the polyploid *Brassica juncea*, and the effects of allopolyploidization on the divergence of gene sequence, structure, function, and expression were assessed. The deduced protein sequences of the four *BjuMYB28* genes showed 76.1–83.1% identity with the *Arabidopsis* MYB28. Phylogenetic analysis revealed that the four BjuMYB28 proteins have evolved via the hybridization and duplication processes forming the *B. juncea* genome (AABB) from *B. rapa* (AA) and *B. nigra* (BB), while retaining high levels of sequence conservation. Mutant complementation and over-expression studies in *A. thaliana* showed that all four *BjuMYB28* genes encode functional MYB28 proteins and resulted in similar aliphatic glucosinolate composition and content. Detailed expression analysis using qRT-PCR assays and promoter-*GUS* lines revealed that the *BjuMYB28* genes have both tissue- and cell-specific expression partitioning in *B. juncea*. The two B-genome origin *BjuMYB28* genes had more abundant transcripts during the early stages of plant development than the A-genome origin genes. However, with the onset of the reproductive phase, expression levels of all four *BjuMYB28* increased significantly, which may be necessary for producing and maintaining high amounts of aliphatic glucosinolates during the later stages of plant development. Taken together, our results suggest that the four *MYB28* genes are differentially expressed and regulated in *B. juncea* to play discrete though overlapping roles in controlling aliphatic glucosinolate biosynthesis.

## Introduction

Glucosinolates are a diverse group of nitrogen- and sulphur-rich secondary metabolites characteristic of the order Capparales, which includes nutritionally important *Brassica* crops and the model plant *Arabidopsis thaliana* (Fahey *et al.*, 2001; Wittstock and Halkier, 2002). Based on the precursor amino acid used, glucosinolates are broadly classified into three major groups namely aliphatic, indolic, and aromatic. The biosynthesis of glucosinolates can be divided into three phases: (i) recruitment of precursor amino acids and side-chain elongation, (ii) formation of the core glucosinolate structure, and (iii) side group modification (Halkier and Gershenzon, 2006). Together with side-chain elongation of the R-group, side-chain modifications generate a wide variety of glucosinolate compounds, with more than 200 structures identified to date (Clarke, 2010).

In recent years, glucosinolates and their breakdown products have been the subject of extensive studies due to their role in defence against pests and pathogens (Halkier and Gershenzon, 2006). Some glucosinolates and their breakdown products have anti-nutritional and goitrogenic properties in seed meal, while others act as anti-carcinogenic compounds in mammals (Fahey *et al.*, 1997; Juge *et al.*, 2007; Cartea and Velasco 2008; Traka and Mithen, 2009). Due to their diverse roles in plant metabolism, animal nutrition, and disease, glucosinolates are a potential target for genetic manipulation in crop improvement programmes.

Our understanding about the genes controlling the complex trait of glucosinolate accumulation has been obtained from various molecular-genetic and ‘omics’ based studies on the model plant *A. thaliana* (Halkier and Gershenzon, 2006; Gigolashvili *et al.*, 2009; Sonderby *et al*., 2010*b*). To date, more than 20 glucosinolate biosynthesis pathway genes have been identified in *Arabidopsis*. Recent reports have confirmed that glucosinolate levels are further controlled by at least six members of subgroup-12 of the R2R3-MYB superfamily. The *Arabidopsis AtMYB28*, *AtMYB29*, and *AtMYB76* genes act as transcriptional regulators of aliphatic glucosinolate biosynthesis (Gigolashvili *et al.*, 2007*b*, 2008; Hirai *et al.*, 2007; Sonderby *et al.*, 2007, 2010*a*), whereas AtMYB34, AtMYB51, and AtMYB122 specifically regulate indolic glucosinolate formation (Celenza *et al.*, 2005; Gigolashvili *et al.*, 2007*a*).

The crops belonging to the genus *Brassica* have been of great economical importance to mankind because of their potential use as vegetables, oilseeds, feed, condiments, fodder, green manure, and even medical treatments. In *Brassica* crops, glucosinolate content and profiles are highly variable and species-specific, with aliphatic glucosinolates (derived from methionine) being the predominant glucosinolates (up to 95% of the total glucosinolates) in seeds (Sodhi *et al.*, 2002). Over the past few decades, there have been ongoing breeding efforts towards the enrichment of beneficial aliphatic glucosinolates (e.g. glucoraphanin) and the reduction of anti-nutritional aliphatic glucosinolates (e.g. progotrin, gluconapin) in these crops. Genetic studies have shown that seed aliphatic glucosinolate content is a quantitative trait, controlled by a variable number of loci in both *B. napus* (Toroser *et al.*, 1995; Uzunova *et al.*, 1995; Howell *et al.*, 2003; Feng *et al.*, 2012) and *B. juncea* (Cheung *et al.*, 1998; Sodhi *et al.*, 2002; Mahmood *et al.*, 2003; Ramchiary *et al.*, 2007; Bisht *et al.*, 2009). Although these reports have identified few genomic regions (QTLs) that control the variability of glucosinolate contents and profiles across *Brassica* species, our understanding of the molecular-genetic mechanism controlling such an economically important trait in *Brassica* species is largely limited at present.

Allopolyploidy is a condition in which a cell has two or more sets of chromosomes derived from two different species. For example, *B. juncea* (2*n*=4*x*=36) is an allotetraploid having a set of chromosomes derived from *B. rapa* (2*n*=2*x*=20) and a set from *B. nigra* (2*n*=2*x*=16). Because of the occurrence of polyploidy and genome-wide rearrangements, the regulation of aliphatic glucosinolate biosynthesis in *Brassica* species is expected to be highly complex compared with that in the closely related diploid *Arabidopsis*. Comparative mapping studies between *Brassica* species and *Arabidopsis* revealed the triplicate nature of diploid *Brassica* genomes and strongly suggested that the extant diploid *Brassica* species have evolved from a common hexaploid ancestor at about 11–12 MYA (Parkin *et al.*, 2005). Further, genomes of the allotetraploid *B. napus* and *B. juncea* are even more complex and are known to retain up to six conserved segments/blocks of the ancestral genome (Schranz *et al.*, 2006; Panjabi *et al.*, 2008). Thus, the existence of multiple homologues of each glucosinolate candidate gene in allotetraploid *Brassica* genomes is expected. Studies on the expression and functional variance of the homologous genes arising from polyploidy are therefore fundamentally important for a better understanding of the complex mechanisms controlling glucosinolate accumulation in these crops. Such an understanding would ultimately allow for the manipulation of specific genes for targeted engineering of glucosinolate accumulation without compromising overall plant fitness.

To date none of the transcriptional regulators of aliphatic glucosinolate biosynthesis genes from any of the *Brassica* crops have been functionally characterized. The isolation of multiple *MYB28* gene homologues from an economically important oilseed crop of the *Brassica* genus, *B. juncea* (AABB genome) as well as from genomes of its two progenitors namely, *B. rapa* (AA genome) and *B. nigra* (BB genome) is reported here. The consequence of polyploidy on gene structure, phylogeny, and gene expression and function has been investigated in detail. Taken together, our results highlight the importance of *MYB28* homologues towards controlling complex glucosinolate traits in polyploid *B. juncea*, which could be utilized towards manipulating the pest resistance, anti-nutritional properties and health benefits of oilseed cultivars of this species.

## Materials and methods

### Plant materials and growth conditions

High glucosinolate cultivars of *B. nigra* (cv. IC257), *B. rapa* (cv. YID1), and *B. juncea* (cv. Varuna) were grown in a growth chamber set at day (22 °C, 10h)/night (15 °C, 14h) cycle, 70% relative humidity, and light intensity <250 µmol m^–2^ s^–1^. Different developmental stages, namely seedling, root, stem, leaf (primary leaf, young, mature, and flag), and silique (10, 20, and 30 d post-anthesis: dpa), were collected, frozen in liquid nitrogen and stored at –80 °C.

*A.* *thaliana* wild-type (ecotype Col-0) and the *AtMYB28* (At5g61420) loss-of-function mutant, BRC_H161b (Beekwilder *et al.*, 2008) were grown in a growth room set at 22 °C under 16/8h light/dark cycle and at 40% relative humidity.

### Isolation of genomic and cDNA sequences of *MYB28* homologues from *B. juncea*

The full-length genomic sequences and the coding sequences of the *BjuMYB28* genes were isolated from *B. juncea* (cv. Varuna) as well as species representing its progenitor genomes namely, *B. nigra* (cv. IC257) and *B. rapa* (cv. YID1).

Total RNA was isolated using the TRI-Reagent (Sigma). DNase (New England Biolabs) treatment was performed on total RNA and about two microgram of RNA was reverse transcribed into cDNA with oligo-dT primers using a first strand cDNA synthesis kit (Applied Biosystems). Full-length coding sequences were obtained by performing 5′ and 3′ RACE using gene-specific primers and SMART^TM^ RACE cDNA amplification kit (Clontech).

For obtaining the genomic sequences, PCR amplification was performed on total genomic DNA using the gene-specific primers designed based on cDNA sequences obtained above. PCR products were cloned into pGEMT-Easy cloning vector (Promega), sequenced and analysed using DNASTAR software (Lasergene). A list of primers used in the current study is summarized in Supplementary Table S1 at *JXB* online.

### Quantitative RT-PCR (qRT-PCR) analysis

The relative expression of glucosinolate pathway genes was analysed by real-time qRT-PCR in an ABI 7900HT Fast Real-time PCR machine (ABI) using a SYBR green protocol. Approximately, two micrograms of total RNA were reverse-transcribed using high capacity first strand cDNA synthesis kit (ABI) according to the manufacturer’s instructions. The *ACTIN2* and *GAPDH* genes were used as endogenous controls (Chandna *et al.*, 2012). Data were analysed from at least three independent sets of biological replicates (separate plant/transgenic lines) with two technical replicates for each. Primers used for qRT-PCR analysis are tabulated in Supplementary Table S1 at *JXB* online.

### Subcellular localization of BjuMYB28 proteins

To generate a Pro_CaMV35S_:BjuMYB28:YFP fusion construct, the coding sequence of *BjuMYB28-2* was cloned into a C-terminal YFP fusion vector pEarlygate101 (Earley *et al.*, 2006). The *AtMYB28* was used as a reference control. Constructs were transformed into onion epidermal cells through a particle delivery system (PDS 1000, Bio-Rad) according to the manufacturer’s instructions. Localization of the BjuMYB28:YFP fusion protein was determined after 48h of incubation in the transformed cells under a confocal laser scanning microscope (Leica).

### Generation of plant transformation constructs

For the development of *BjuMYB28* over-expression constructs, the coding sequences of the *BjuMYB28* genes were cloned into the pPZP200 binary vector (Hajdukiewicz *et al.*, 1994) under the control of a CaMV 35S promoter along with the *bar* gene as the plant selection marker. The *BjuMYB28* over-expression constructs were transformed into a homozygous loss-of-function mutant of *AtMYB28* (BRC_H161b) and the wild-type (Col-0) genetic background.

For the generation of Pro_BjuMYB28_:*uidA* constructs, the promoter regions (~1kb) of the four *BjuMYB28* genes were isolated from genomic DNA of *B. juncea* by a genome walking protocol (Universal Genome Walker Kit, Clontech) and cloned into the gateway binary vector pMDC164 upstream of the *uidA* gene. Histochemical analysis of GUS reporter protein in independent single-copy transgenic lines (in the T_3_ generation) was performed according to the protocol described by Jefferson *et al.* (1987). The GUS staining patterns at different stages of development were recorded under a Zoom Stereo microscope (SMZ-U, Nikon).

The different constructs used in the current study were transformed into *A. tumefaciens* strain GV3101 by the freeze–thaw method and subsequently into *A. thaliana* by the floral dip method (Clough and Bent, 1998). **S**election of transgenic plants was performed on appropriate antibiotics/selection agents. Both mutant-complemented and over-expression lines were selected on the herbicide Basta (at 120mg l^–1^ of phosphinothricin, 3–4 sprays at 1 d intervals). The Pro_BjuMYB28_:*uidA* lines were selected on hygromycin (20mg l^–1^).

### HPLC analysis for glucosinolate estimation

The extraction and quantification of glucosinolates was performed on leaves of 25-d-old *Arabidopsis* plants by HPLC as per the protocols described earlier by Brown *et al.* (2003). Briefly, glucosinolates were extracted twice in 70% methanol after adding 6mM glucotropaeolin (Applichem) as the internal standard. Samples were loaded on DEAE Sephadex A25 columns and desulphated overnight using purified sulphatase (type H1 from *Helix pomatia*) prior to HPLC. Concentrations of individual glucosinolates were calculated in nmol mg^–1^ dry weight relative to the area of the internal standard peak using the respective response factors reported earlier (Brown *et al.*, 2003). For each *BjuMYB28* construct, at least two experimental replicates of two independent transgenic lines were used for the glucosinolate analyses. The following glucosinolates were detected; 4-methylsulphinylbutyl-glucosinolate (4MSOB), 3-methylsulphinylpropyl-glucosinolate (3MSOP), 5-methylsulphinylpentyl-glucosinolate (5MSOP), 4-methylthiobutyl-glucosinolate (4MTB), 7-methylsulphinylheptyl-glucosinolate (7MSOH), 8-methylsulphinyloctyl-glucosinolate (8MSOO), 6-methylsulphinylhexyl-glucosinolate (6MSOH), indol-3-ylmethyl-glucosinolate (I3M), 1-methoxyindol-3-ylmethyl-glucosinolate (1MOI3M), and 4-methoxyindol-3-ylmethyl-glucosinolate (4MOI3M).

### Sequence and phylogenetic analysis

The coding sequences and amino acids of the *BjuMYB28* genes were predicted using the software DNASTAR (Lasergene). Multiple sequence alignments of coding sequences and the deduced amino acid sequences of *MYB28* from *Arabidopsis* and *Brassica* species were performed using ClustalW. Percentage similarities among MYB28 homologues were calculated using the MegAlign module of DNASTAR.

The BLASTX search of AtMYB28 and four BjuMYB28 coding sequences was performed on the publicly available database at NCBI (http://blast.ncbi.nlm.nih.gov/Blast.cgi) and phytozome (http://phytozome.net). Full-length coding sequences showing high percentage similarity scores and sequence coverage were obtained. Evolutionary history was inferred using the Maximum Likelihood method (Saitou and Nei, 1987) based on the JTT matrix-based model in MEGA5 (Tamura *et al.*, 2011). MYB28 like sequences reported from the following genomes were used for the analysis: *Arabidopsis thaliana* (AtMYB28, AtMYB29, AtMYB76); *Arabidopsis lyrata* (Aly_950898, Aly_487604, Aly_892702); *Capsella rubella* (Cru_10027861m, Cru_10001328m); *Thellungiella halophila* (Tha_v10004407m, Tha_v10013952m); *Brassica rapa* (Bra035929, Bra012961, Bra029311), and *Brassica oleracea* (BolC.MYB28.1/ADK38583, BolC.MYB28.2/CBI71385). The deduced proteins from the *B. rapa* (BraA.MYB28.1, BraA.MYB28.2); *B. nigra* (BniB.MYB28.1, BniB.MYB28.2), and *B. juncea* (BjuMYB28-1, -2, -3, and -4) genomes were also used.

### Statistical analysis

Data from different experimental sets were analysed for statistical significance using one-way ANOVA applying Fishers LSD or Tukey’s *post hoc* test. A *P* value <0.05 was considered as significant.

### Accession numbers

The sequences isolated in the current study were submitted to GenBank. Accession nos. JQ666166 (BjuMYB28-1 CDS), JQ666167 (BjuMYB28-2 CDS), JQ666168 (BjuMYB28-3 CDS), JQ666169 (BjuMYB28-4 CDS), JQ700565 (BjuMYB28-1 full-length gene), JQ700566 (BjuMYB28-2 full-length gene), JQ700567 (BjuMYB28-3 full-length gene), JQ700568 (BjuMYB28-4 full-length gene), JX947841 (BniMYB28-1 CDS), and JX947842 (BniMYB28-2 CDS).

## Results

### Isolation of *MYB28* homologues from *B. juncea* and its progenitor species

Degenerate primers (see Supplementary Table S1 at *JXB* online) based on the reported sequence of *A. thaliana* AtMYB28 (At5g61420) protein were used to isolate partial cDNA sequences of *MYB28* genes from *B. juncea*. A total of four partial *MYB28*-like sequences were isolated from this polyploid. Using a combination of 5′ and 3′ RACE strategy, four full-length *BjuMYB28* sequences [designated as *BjuMYB28-1* (Accession no. JQ666166), *BjuMYB28-2* (JQ666167), *BjuMYB28-3* (JQ666168), and *BjuMYB28-4* (JQ666169)] were identified and confirmed with multiple amplifications from different tissue types (Table 1). The nucleotide sequences of these cDNAs showed 79.8–89.4% sequence identity in their coding regions (see Supplementary Fig. S1 and Supplementary Table S2 at *JXB* online). The open reading frames of the four *BjuMYB28* sequences varied from 1053–1095bp, encoding proteins of 351–365 amino acids (Augustine *et al.*, 2013).

**Table 1.** DNA sequence summary of the BjuMYB28 genes identified in the current study

S. no.	Gene	Gene (bp)	CDS (bp)	Protein (aa)	No. of exons (sizes in bp)	No. of introns (sizes in bp)	3′UTR	Promoter (5′GW)	Progenitor *Brassica* genome
1.	*AtMYB28*	1321	1101	367	3 (133, 130, 838)	2 (80, 140)	324	–	–
2.	*BjuB.MYB28.1 (BjuMYB28-1)*	1630	1053	351	3 (133, 130, 790)	2 (72, 505)	180	1040	B
3.	*BjuB.MYB28.2* (*BjuMYB28-2*)	1315	1095	365	3 (133, 130, 832)	2 (82, 138)	180	1275	B
4.	*BjuA.MYB28.1* (*BjuMYB28-3*)	1508	1065	355	3 (133, 130, 802)	2 (79, 364)	240	1100	A
5.	*BjuA.MYB28.2* (BjuMYB28-4)	1350	1065	355	3 (133, 130, 802)	2 (89, 196)	174	2400	A

To determine the genomic structure of the four *BjuMYB28* sequences, PCR amplification on total genomic DNA of *B. juncea* was performed using sequence-specific primers. The four genomic sequences varied in size, ranging from 1314–1630bp in length (Table 1; Accession nos. JQ700565–68). Comparison of the four *BjuMYB28* cDNAs with their corresponding genomic sequences showed that all four genes consisted of two introns and three exons, with introns found to be more divergent in size and composition as compared to the coding regions (see Supplementary Fig. S2 and Supplementary Table S3 at *JXB* online).

*B. juncea* is a natural allopolyploid species (AABB genome) formed from hybridization of *B. rapa* (AA genome) and *B. nigra* (BB genome). In order to assign the progenitor sub-genome to each *BjuMYB28*, *MYB28* homologues from *B. rapa* and *B. nigra* were also isolated. Two *MYB28* sequences could be isolated from both *B. rapa* (named as *BraA.MYB28.1* and *BraA.MYB28.2*) and *B. nigra* (named as *BniB.MYB28.1* and *BniB.MYB28.2*), representing the paralogous gene pair from each progenitor genome. Sequence analysis confirmed that two *BjuMYB28* genes namely, *BjuMYB28-1* and *BjuMYB28-2* were similar to the B-genome paralogues (see Supplementary Fig. S1 at *JXB* online), and so will henceforth be referred to as *BjuB.MYB28.1* and *BjuB.MYB28.2*, respectively (Table 1). Similarly, the nucleotide sequences of the remaining two *B. juncea* genes (*BjuMYB28-3*, and *BjuMYB28-4*) were highly similar to those of A-genome paralogues, and so will be referred to as *BjuA.MYB28.1* and *BjuA.MYB28.2*, respectively. In general, the coding sequences of the *MYB28* genes isolated from the species containing the progenitor A- and B-genomes showed very high levels of sequence identity (99.7–99.9%) with their corresponding *MYB28* homologues from *B. juncea* (see Supplementary Fig. S1 and Supplementary Table S2 at *JXB* online). Our data are in accordance with the reported allopolyploid origin of *B. juncea* (Panjabi *et al.*, 2008), wherein both progenitor genomes have contributed a duplicated *MYB28* gene pair (paralogues).

### Sequence analysis and phylogeny of BjuMYB28 proteins

**Fig. 1.**
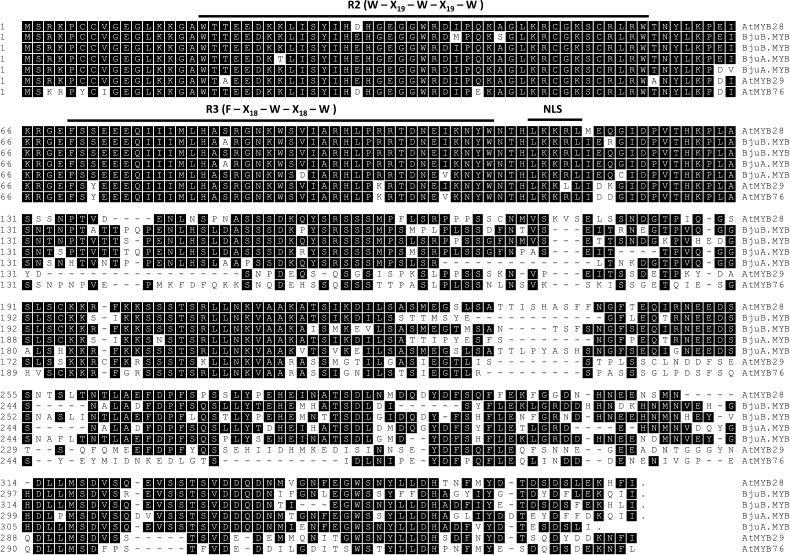
Amino acid sequence alignment of BjuMYB28 proteins. The sequence alignment of the four BjuMYB28 proteins with the known aliphatic glucosinolate-regulating MYB proteins of *A. thaliana* namely, AtMYB28, AtMYB29, and AtMYB76 was performed using Clustal W. Consensus sequences for R2 and R3 domains (Dubos *et al.*, 2010) are marked as solid lines. The putative nuclear localization signal (LKKRL) is also marked (NLS).

In order to investigate the evolutionary origin of the four *BjuMYB28* genes further, an amino acid sequence alignment of the deduced BjuMYB28 proteins was created with the known MYB aliphatic glucosinolate regulators from *A. thaliana*. The biosynthesis of aliphatic glucosinolates in *A. thaliana* is regulated by three closely related members of subgroup-12 of the R2R3-MYB superfamily namely AtMYB28, AtMYB29 and AtMYB76 (Gigolashvili *et al.*, 2007*b*, 2008; Hirai *et al.*, 2007; Sonderby *et al.*, 2007, 2010*a*). The deduced BjuMYB28 proteins shared maximum identity with the AtMYB28 protein (72.8–82.3%) among the three aliphatic glucosinolate regulators of *Arabidopsis* (Table 2). The deduced protein sequences of the four *BjuMYB28* sequences were 74.9–86.6% identical with each other. Amino acid sequence alignment confirmed the presence of two imperfect sequence repeats (R2R3 repeat) at the N-terminal region (Fig. 1). Predicted signature sequences for both R2 (-W-X_19_-W-X_19_-W-) and R3 (-F-X_18_-W-X_18_-W-) repeats showed a high level of sequence conservation among BjuMYB28 proteins. In contrast to the highly conserved N-terminal DNA-binding MYB domains, the downstream C-terminal region of BjuMYB28 proteins showed homology only in patches (Fig. 1). The structural divergence of BjuMYB28 proteins might have consequences for their differential gene function(s) leading to loss or silencing, maintenance of ancestral function, or functional divergence either through sub-functionalization or neo-functionalization (Lynch and Conery, 2000; Adams, 2007).

**Table 2.** Amino acid sequence identity (%) of BjuMYB28 proteins with known aliphatic glucosinolate regulators belonging to subgroup-12 of the R2R3-MYB superfamily of *A. thaliana*

	AtMYB28	BjuB.MYB28.1	BjuB.MYB28.2	BjuA.MYB28.1	BjuA.MYB28.2	AtMYB29	AtMYB76
		(BjuMYB28-1)	(BjuMYB28-2)	(BjuMYB28-3)	(BjuMYB28-4)		
AtMYB28	***	72.8	82.3	74.9	80.4	59.1	55.9
BjuB.MYB28.1		***	80.1	86.6	74.9	57.1	55.9
(BjuMYB28-1)							
BjuB.MYB28.2			***	77.5	81.1	65.2	59.8
(BjuMYB28-2)							
BjuA.MYB28.1				***	76.6	59.5	55.9
(BjuMYB28-3)							
BjuA.MYB28.2 (BjuMYB28-4)					***	60.7	56.5
AtMYB29						***	66.1
AtMYB76							***

**Fig. 2.**
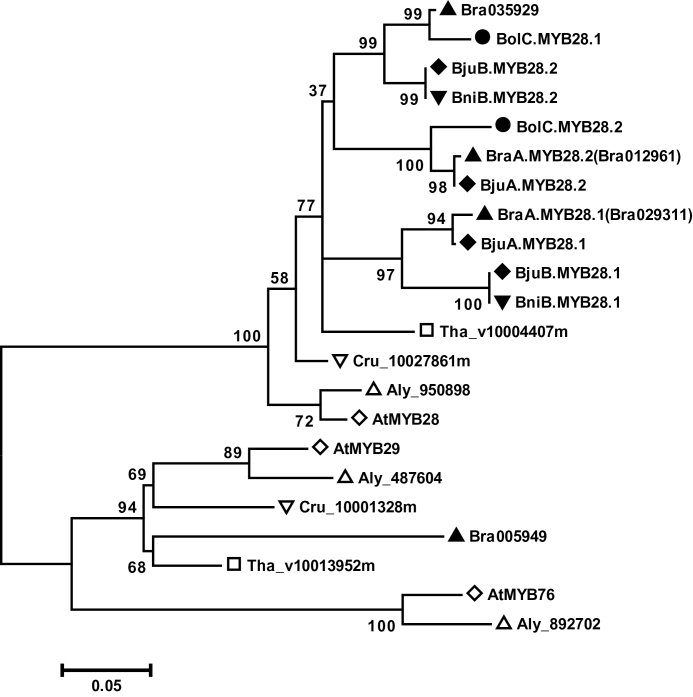
Evolutionary relationships of BjuMYB28 proteins. Phylogenetic analysis of BjuMYB28 proteins with the MYB proteins involved in aliphatic glucosinolate biosynthesis from *A. thaliana* (At, open diamond), *A. lyrata* (Ala, open triangle), *C. rubella* (Cru, open inverted triangle), *T. halophila* (Tha, open square), *B. rapa* (Bra, closed triangle), *B. nigra* (Bni, closed inverted triangle), *B. oleracea* (Bol, closed circle), and *B. juncea* (Bju, closed diamond) genomes was performed using the MEGA5 program (Tamura *et al.*, 2011). The evolutionary history was inferred using the maximum likelihood method (Saitou and Nei, 1987). The percentage of replicate trees in which the associated proteins clustered together in the bootstrap test (1000 replicates) is shown next to the branches. The tree is drawn to scale, with branch lengths measured in the number of substitutions per site.

The high level of amino acid identity and domain conservation indicated that the four BjuMYB28 proteins are closely related. Their evolutionary relationships vis-à-vis the MYB28 proteins isolated from the species containing the two progenitor genomes of *B. juncea* were compared with publicly reported MYB28-like sequences from other plant genomes. On a maximum likelihood tree, the four BjuMYB28 proteins were grouped together with the AtMYB28 protein into a distinct MYB28 sub-group with high bootstrap support; AtMYB29 along with AtMYB76 was found in a separate subgroup (Fig. 2). All four BjuMYB28 proteins shared a close evolutionary ancestry with MYB28-like sequences from *Brassica* species, particularly those obtained from the species containing the two progenitor genomes. For example, the B-genome-specific MYB28 proteins from *B. juncea* grouped with *B. nigra* proteins with very high bootstrap scores. Similarly, the A-genome specific MYB28 proteins of *B. juncea* grouped nicely with *B. rapa* sequences (including the recently reported sequences, Bra029311 and Bra012961). The phylogentic analysis thus clearly revealed that the four BjuMYB28 proteins are evolutionary conserved and have evolved via duplication (paralogues) and hybridization (homeologues) of two relatively simpler *Brassica* genomes, while retaining a very high level of sequence conservation following allo-polyplodization of A- and B-genomes.

### *BjuMYB28* genes encode functional MYB28 proteins regulating the accumulation of aliphatic glucosinolates

**Fig. 3.**
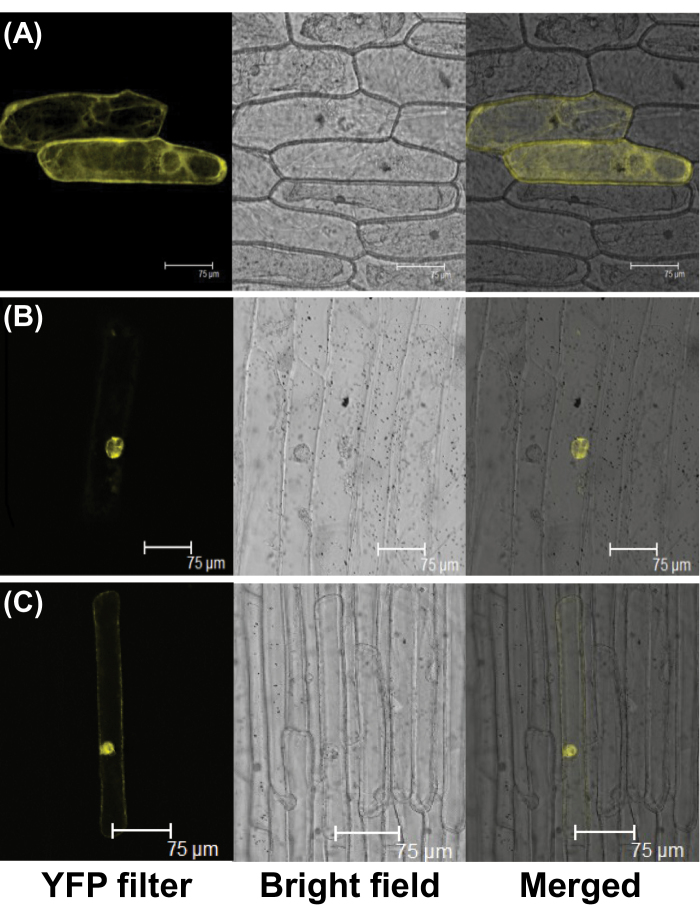
Subcellular localization of BjuMYB28-YFP fusion constructs in onion epidermal cells. The YFP filter, bright field, and merged images of (A) the YFP positive control vector, (B) BjuMYB28-2:YFP, and (C) AtMYB28:YFP fusion proteins are shown.

BjuMYB28 proteins share a close evolutionary relationship to the *Arabidopsis* AtMYB28 protein, a member of R2R3-MYB transcription factor family (Gigolashvili *et al.*, 2007*b*). Although BjuMYB28 proteins lack the typical nuclear localization signal when queried using the PredictProtein software (http://www.predictprotein.org/), a conserved SV40-type putative nuclear localization motif (LKKRL; van der Krol and Chua, 1991) adjacent to the R3 repeat of these proteins was observed (Fig. 1). Thus, to investigate their subcellular localization, a representative BjuMYB28 protein (BjuMYB28-2) was selected and the Pro_35S_:BjuMYB28:YFP construct was introduced into onion epidermal cells by particle bombardment. The transiently transformed cells showed a strong yellow fluorescence signal in the nucleus, thus demonstrating that the BjuMYB28 are predominantly nuclear localized proteins, similar to what was observed for the *Arabidopsis* AtMYB28 protein (Fig. 3).

In the absence of reverse genetics tools in the allopolyploid *B. juncea*, the functional contribution of each *BjuMYB28* towards controlling the glucosinolate pool and profile was tested in the closest model system, *A. thaliana*. All four *BjuMYB28* genes (under the control of the constitutive CaMV 35S promoter) were over-expressed in two different genetic backgrounds of *A. thaliana*, (i) the homozygous BRC_H161b, a *myb28* knock-down T-DNA insertion mutant, and (ii) the wild-type (Col-0). Two independent homozygous lines of each *BjuMYB28* were analysed for total as well as individual glucosinolate fractions in 4-week-old rosette leaves.

**Fig. 4.**
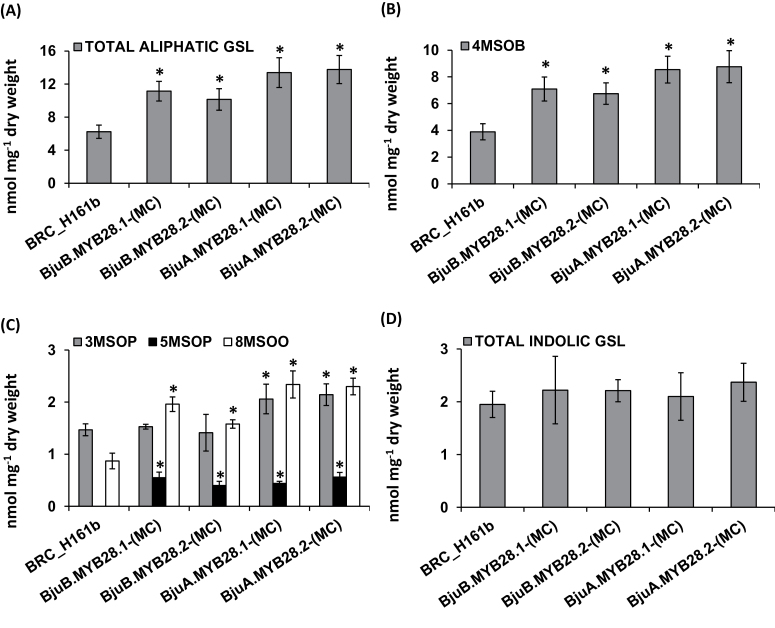
Functional complementation analysis of *BjuMYB28* genes in the *Arabidopsis myb28* mutant (BRC_H161b). Glucosinolate accumulation in the rosette leaves of *Arabidopsis myb28* mutant over-expressing *BjuMYB28* genes. The glucosinolate content and profile (in nmol mg^–1^ dry weight) was determined in 25-d-old rosette leaves. The individual graphs show the accumulation of (A) total aliphatic glucosinolates (GSLs); (B) the predominant GSL, 4MSOB; (C) short and long chain GSLs (3MSOP, 5MSOP, 8MSOO); and (D) total indolic glucosinolate. Two independent mutant-complemented lines for each *BjuMYB28* gene were analysed and the average foliar glucosinolates are represented along with their standard errors. Asterisks indicate significant differences in glucosinolate content compared with the *Arabidopsis* mutant background (*P* <0.05, in Fishers LSD test determined by ANOVA). Abbreviations: 4-methylsulphinylbutyl-glucosinolate (4MSOB), 3-methylsulphinylpropyl-glucosinolate (3MSOP), 5-methylsulphinylpentyl-glucosinolate (5MSOP), 8-methylsulphinyloctyl-glucosinolate (8MSOO).

The functional complementation of the *BjuMYB28* genes elevated the accumulation of total aliphatic glucosinolates 1.6–2.2-fold compared with the mutant BRC_H161b line (Fig. 4A). Both short- and long-chain aliphatic glucosinolates were found to be elevated in these lines (Fig. 4B, C). The level of 4MSOB, the major glucosinolate present in *Arabidopsis*, was found to be increased by 1.7–2.2-fold. The content of other short-chain aliphatic glucosinolates, like 3MSOP and 5MSOP, was also found to be increased by up to 1.5-fold. Accumulation of the long-chain aliphatic glucosinolate 8MSOO was increased from 1.8–2.7-fold compared with the mutant line (Fig. 4C). The indolic glucosinolates (I3M, 1MOI3M and 4MOI3M) of complemented mutant lines were found to be unaltered in content (Fig. 4D). Thus mutant complementation analysis in *A. thaliana* clearly suggested that all four *BjuMYB28* genes specifically regulate the aliphatic glucosinolate pools without altering the non-aliphatic glucosinolate levels.

**Fig. 5.**
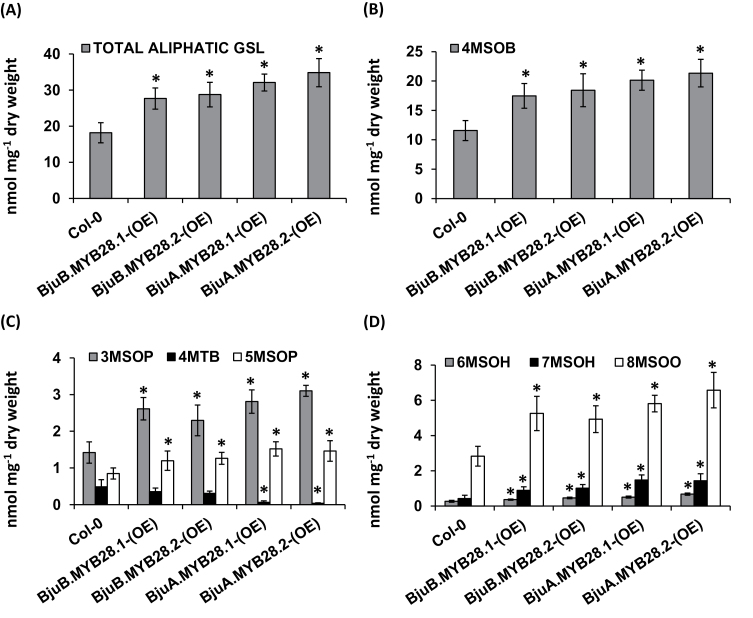
Glucosinolate accumulation in rosette leaves of *BjuMYB28* over-expressing *Arabidopsis* lines (Col-0). The glucosinolate content and profile (in nmol mg^–1^ dry weight) was determined in 25-d-old rosette leaves. The individual graphs show the accumulation of (A) total aliphatic glucosinolates (GSLs), (B) the predominant GSL, 4MSOB, (C) short chain aliphatic glucosinolates including 3MSOP, 4MTB, and 5MSOP, and (D) long-chain aliphatic glucosinolates including 6MSOH, 7MSOH, and 8MSOO. Abbreviations used are given in the Materials and methods. At least two independent over-expression lines for each *BjuMYB28* were analysed and the average foliar glucosinolate are represented together with their standard error. Asterisks indicate significant differences in glucosinolate content compared with the *Arabidopsis* Col-0 wild-type background (*P* <0.05, in Fishers LSD test determined by ANOVA). Abbreviations: 4-methylsulphinylbutyl-glucosinolate (4MSOB), 3-methylsulphinylpropyl-glucosinolate (3MSOP), 4-methylthiobutyl-glucosinolate (4MTB), 5-methylsulphinylpentyl-glucosinolate (5MSOP), 6-methylsulphinylhexyl-glucosinolate (6MSOH), 7-methylsulphinylheptyl-glucosinolate (7MSOH), 8-methylsulphinyloctyl-glucosinolate (8MSOO).

The functional divergence of *BjuMYB28* genes in controlling the levels of aliphatic glucosinolate content and profile was assessed by generating over-expression lines for these homologues in the *A. thaliana* wild-type ecotype Col-0. Total, as well as individual, glucosinolate levels of two over-expression lines of each *BjuMYB28* construct were analysed and compared with the wild-type control. As shown in Fig. 5A, *BjuMYB28* over-expression provided an approximately 1.5–1.9-fold increase in leaf aliphatic glucosinolate accumulation compared with wild-type plants. Levels of 4MSOB, the major glucosinolate present in *Arabidopsis*, were found to be increased by 1.5–1.8-fold (Fig. 5B). Concomitantly, the level of 4MTB (precursor of 4MSOB), showed a significant reduction in over-expression lines for the two A-genome specific *BjuMYB28* genes, thereby reflecting an increased flux of 4C-glucosinolate towards 4MSOB. The contents of short-chain aliphatic glucosinolates like 3MSOP and 5MSOP were also found to be increased up to 2.2- and 1.8-fold, respectively (Fig. 5C). Accumulation of long-chain aliphatic glucosinolates namely 6MSOH, 7MSOH, and 8MSOO, was also increased by 1.3–3.5-fold compared with the wild-type plants (Fig. 5D). Contents of indolic glucosinolates such as I3M, 1MOI3M, and 4MOI3M were found to be unaltered (see Supplementary Fig. S3 at *JXB* online).

The increased glucosinolate accumulation in transgenic lines correlated with increased mRNA levels of the glucosinolate pathway genes of *A. thaliana*. As shown in Supplementary Fig. S4 at *JXB* online, the expression of genes involved in both side-chain elongation (*MAM1, MAM3*) and core biosynthetic steps (*CYP79F1*, *CYP79F2, CYP83A1*, *AtST5b*, *AtST5c*) of the aliphatic glucosinolate biosynthesis pathway were considerably increased in both mutant complementation and over-expression lines compared with their respective backgrounds. The *BjuMYB28* genes tested caused up-regulation of all the aliphatic glucosinolate biosynthetic genes tested, although at variable levels.

Over-expression of *BjuMYB28* genes in both wild-type and mutant backgrounds of *Arabidopsis* showed no visible effects on seed germination, plant growth or development. These lines grew normally and did not show any significant differences in response to different abiotic stress conditions (such as salt, heat, dehydration, cold) compared with their corresponding reference backgrounds (data not shown). Thus, both mutant complementation and over-expression studies clearly demonstrated that all four *BjuMYB28* genes encode functional BjuMYB28 proteins regulating aliphatic glucosinolate pools.

### The four *BjuMYB28* genes exhibit overlapping but distinct expression profiles in *B. juncea*

Genome polyploidy events are often associated with variable expression of the homeologous gene pairs within the genome. The expression profiles of *MYB28* genes were therefore analysed at different developmental stages of *B. juncea* as well as in progenitor species. The efficiency and specificity of gene-specific *BjuMYB28* primer pairs was initially ascertained using a 10-fold serial dilution of the corresponding plasmid DNA. A linear correlation coefficient (*R* ^2^) of 0.99 and above was observed over a 100 000-fold dilution range, which reflected the high efficiency of each primer pair (see Supplementary Table S4 at *JXB* online). The expression profiles of *MYB28* genes across various developmental stages (or tissue types) of the species representing the three *Brassica* genomes were compared with endogenous control genes.

**Fig. 6.**
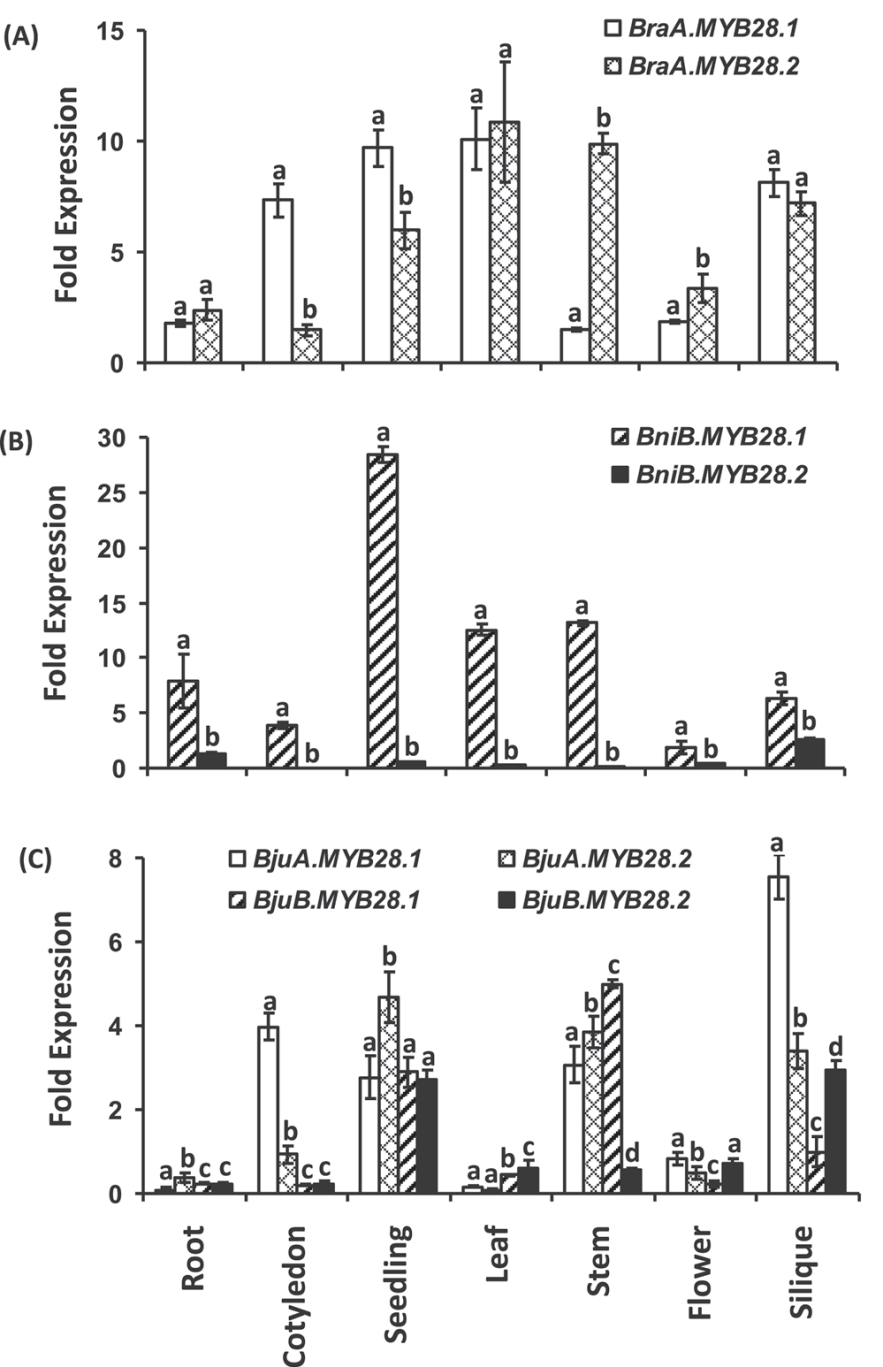
Expression profile of *BjuMYB28* genes in organs of *B. juncea* and its progenitor genomes. Expression profile of *MYB28* genes across various developmental stages/tissue types in (A) *B. rapa* (A genome), (B) *B. nigra* (B genome) and *B. juncea* (AB genome). The stages are defined as: root (15 d), cotyledons, seedling (7 d), leaf (15 d), stem (30 d), flowers (open), silique (10 d post-anthesis). Real-time quantitative PCR (qRT-PCR) was conducted and expression values across different tissue types were normalized against *Actin* gene expression (set at 100). Each bar represents the mean (± standard error) of three independent biological replicates. Different letters on the top indicate significant differences at *P* <0.05 in Tukey’s *post hoc* test.

The expression profiles of *MYB28* genes were investigated first in the progenitor species of *B. juncea* namely, *B. rapa* (A-genome) and *B. nigra* (B-genome), each harbouring two *MYB28* paralogues. Both A-genome specific *MYB28* paralogues showed very comparable expression profiles when tested across developing stages of *B. rapa* (Fig. 6A). The two *BraMYB28* paralogues were abundantly expressed in glucosinolate synthesizing tissues such as seedling, leaf, and silique, whereas roots and flowers showed less accumulation of these transcripts. By contrast, when the expression profile of *MYB28* homologues was studied in *B. nigra*, differential expression of the two *BniMYB28* transcripts was observed. The *BniB.MYB28.1* gene was highly expressed across all the developing stages, particularly in seedlings and roots, whereas the *BniB.MYB28.2* transcript showed reduced expression in all the tested tissues (Fig. 6B).

In order to determine whether there is a bias in transcript levels from one of the two genomes in the polyploid *B. juncea*, qRT-PCR analysis was performed for all four *BjuMYB28* in the same tissue types. All four *BjuMYB28* genes were expressed in most of the tissue types (Fig. 6C). Higher levels of expression were observed in seedling, stem, and siliques compared with root and primary leaf tissues. The expression profile of A-genome-specific *BjuMYB28* genes was almost similar to that observed in *B. rapa* whereas the two B-genome specific homologues showed altered and reduced expression in *B. juncea* in most of the tissue types tested (Fig. 6). In general, the cumulative abundance of the A-genome-specific homologues was higher than the B-genome-specific homologues in most of the tissue types tested.

**Fig. 7.**
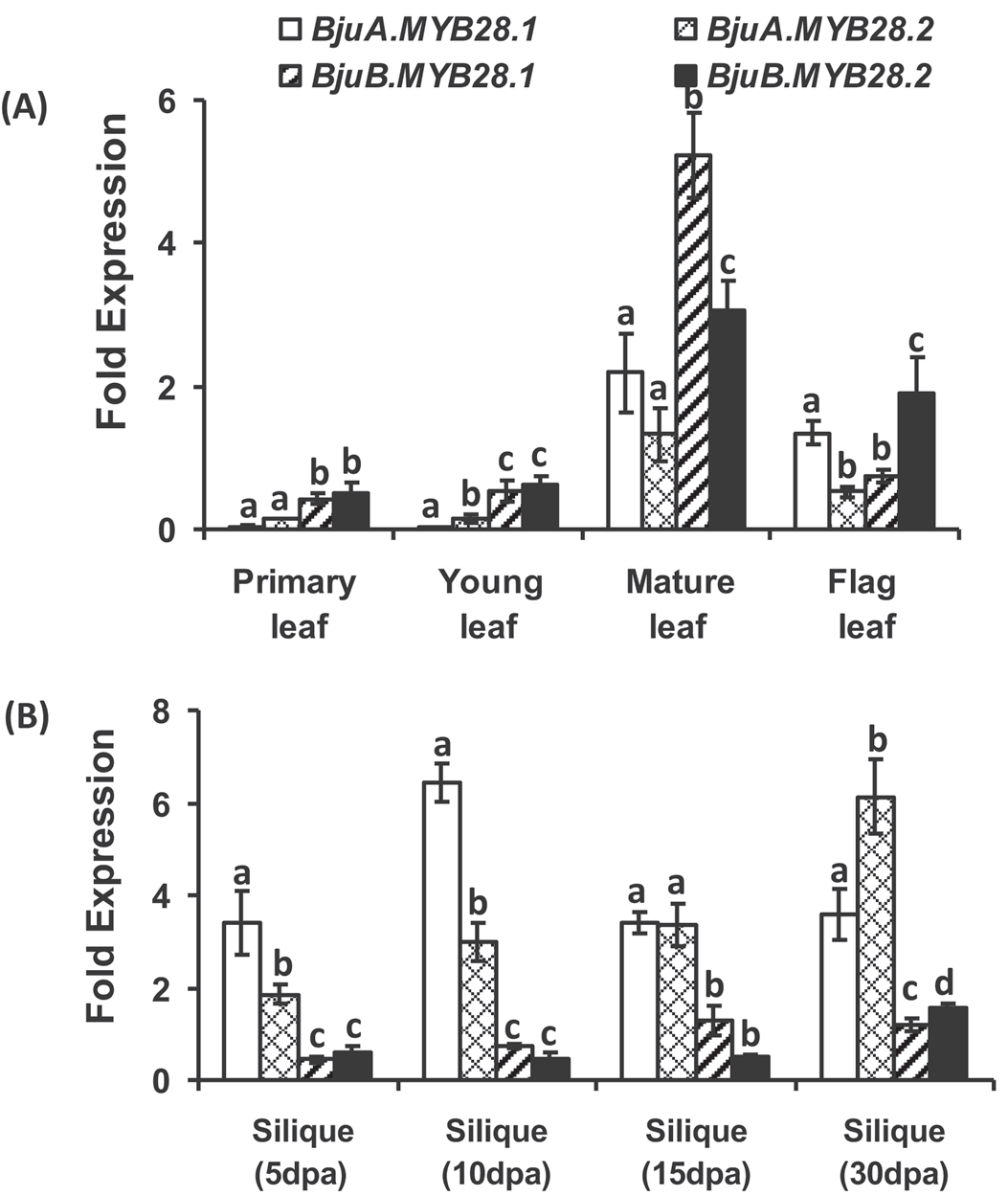
Expression profile of *BjuMYB28* genes in different tissue types of *B. juncea*. Expression of the *BjuMYB28* genes was performed across *B. juncea* (A) developing leaf stages and (B) developing stages of siliques. The stages are defined as: primary leaf (15 d), young leaf (30 d), mature leaf (60 d), flag (inflorescence) leaf, silique 5, 10, 15, and 30 dpa (days post-anthesis). qRT-PCR was conducted and expression values across different tissue types were normalized against *B. juncea Actin* gene expression (set at 100). Each bar represents the mean (±standard error) of three independent biological replicates. Different letters on the top indicate significant differences at *P* <0.05 in Tukey’s *post hoc* test.

Detailed expression analysis of A- and B-genome-specific *BjuMYB28* genes was further performed at different developmental stages of leaves and siliques in *B. juncea*, the tissues where the biosynthesis of glucosinolates largely occurs. All four *BjuMYB28* genes were expressed throughout leaf development, but had lower transcript abundance during the younger stages. However, the B-genome-specific *BjuMYB28* genes had relatively higher transcript accumulation in the primary and young leaves with the onset of the reproductive phase. A significantly higher accumulation of all four *BjuMYB28* transcripts was observed in the mature and inflorescence leaves (Fig. 7A). During the developing stages of the siliques (5, 10, 15, and 30 d post-anthesis), expression of both A-genome-specific *BjuMYB28* homologues was comparably higher (Fig. 7B). The data clearly reflect the distinct expression of A- and B-genome-specific *BjuMYB28* genes across the developmental stages of allopolyploid *B. juncea*.

### Histochemical analysis of Pro_BjuMYB28_:*uidA* lines in *Arabidopsis* confirms differential regulation of *BjuMYB28*

In order to confirm the tissue- as well as cell-specific expression of A- and B-genome-specific *BjuMYB28*, Pro_BjuMYB28_:*uidA* lines of all four *BjuMYB28* promoters were developed in the closely related *A. thaliana*. Approximately 1kb upstream of the ATG (translation start codon) of the four *BjuMYB28* genes was isolated from the *B. juncea* genome using a 5′ genome walking protocol. The 5′ upstream regions showed a much lower level of sequence identity compared with the protein-coding regions (see Supplementary Fig. S5 and Supplementary Table S5 at *JXB* online). Two independent single-copy transgenic lines of each *BjuMYB28* promoter were used for detailed GUS histochemical analysis in the homozygous T_3_ generation.

**Fig. 8.**
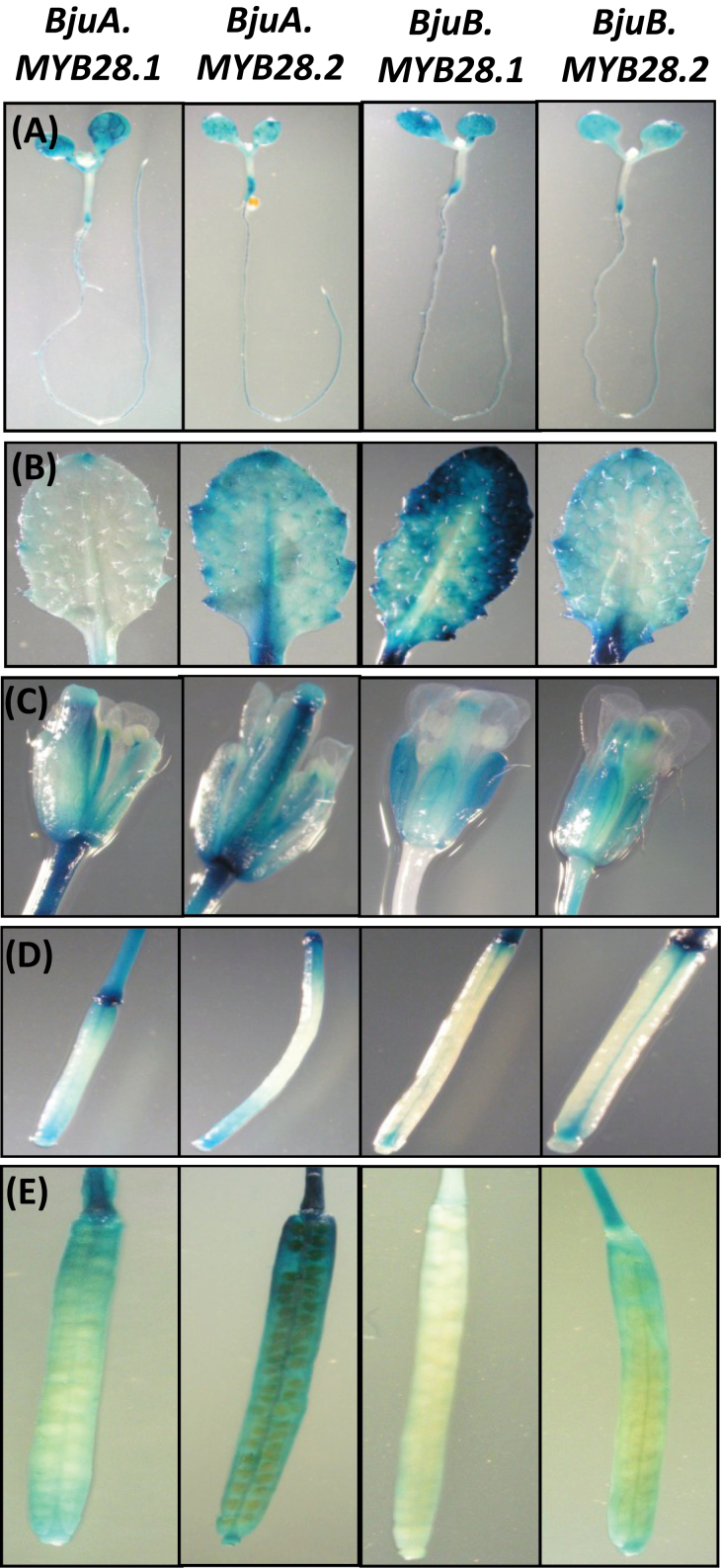
Histochemical GUS staining of Promoter_BjuMYB28_ *-GUS* transgenic *Arabidopsis* lines during different developmental stages and wounding. (A) Two-week old seedlings, (B) 4-week-old rosette leaves, (C) flowers, (D) immature silique, (E) mature silique, and (E) cut ends of leaf of *Pro:GUS* plants for all four *BjuMYB28* homologues. Two independent single copy transgenic lines of each *BjuMYB28* homologues were tested for the GUS histochemical assay in the T_3_ generation.

Reporter gene expression in transgenic *Arabidopsis* lines revealed that all four *BjuMYB28* homologues showed overlapping but distinct expression patterns during the developing stages of *A. thaliana* (Fig. 8). Prominent GUS staining was detected in seedlings, mature rosette leaves, flowers, and siliques of Pro_BjuMYB28_:*uidA* transgenic lines. Among all four promoters, those of *BjuA.MYB28.1* and *BjuA.MYB28.2* exhibited somewhat higher GUS staining than those of the other *MYB28* genes in all floral organs including pistil, anther, and receptacle tissues as well as in the flower stalk (Fig. 8C). Similarly, a stronger reporter gene expression in the developing siliques was also observed for these promoters (Fig. 8D). Thus a higher GUS activity of A-genome versus B-genome-specific *BjuMYB28* promoters was detected in the reproductive tissue. A trace amount of GUS activity was detected in mature roots, senescent leaves, and seeds for all the four *BjuMYB28* promoters tested.

In the rosette leaves of *Arabidopsis*, the *BjuMYB28* promoters showed cell-specific GUS activity. The *BjuB.MYB28.1* and *BjuB.MYB28.2* promoters showed maximal activity in the edges and lamina of the leaves, respectively, whereas the activity of the *BjuA.MYB28.2* promoter was observed in the mid-vein, primary and secondary veins and also towards the leaf edges (Fig. 8B). The *BjuA.MYB28.1* promoter, however, showed traces of GUS staining in the leaf edges. This non-uniform and cell-specific expression pattern of *BjuMYB28* genes within leaves might have important implications for the regulation of glucosinolate content and profile across different regions of the leaf. Thus, the GUS histochemical data obtained using the Pro_BjuMYB28_:*uidA Arabidopsis* lines confirmed that the four *BjuMYB28* promoters have overlapping but distinct cell and tissue expression patterns.

## Discussion

The economically and nutritionally important *Brassica* crops, such as oilseed rape (*B. rapa*, *B. napus*), cabbage (*B. oleracea*), and mustard (*B. juncea*) are rich sources of glucosinolates, particularly the aliphatic glucosinolates. In this study, the isolation, characterization, expression, and functional analysis of *MYB28* gene family homologues, major regulators of aliphatic glucosinolate biosynthesis, is reported in *B. juncea*. The four *BjuMYB28* genes exhibited different, but overlapping tissue- and cell-specific expression patterns, suggesting a co-ordinated role towards controlling aliphatic glucosinolate accumulation in *B. juncea*.

### Genome origins and phylogeny of *B. juncea MYB28* genes

In this study, four *MYB28* homologues were identified in *B. juncea* and it was possible to determine the genome origin of each homologue based on the sequence identity with the *MYB28* genes from *B. rapa* and *B. nigra*. Two *BjuMYB28* genes (*BjuA.MYB28.1* and *BjuA.MYB28.2*) showed the greatest sequence similarity with *MYB28* sequences from *B. rapa* and, therefore, are believed to originate from the A-genome, whereas the remaining two *BjuMYB28* (*BjuB.MYB28.1* and *BjuB.MYB28.2*) were B-genome-specific. The multiplicity of *BjuMYB28* corresponds well with the allotetraploid genome architecture of *B. juncea*, wherein two genes each are derived from the ‘A’ (*B. rapa*) and ‘B’ (*B. nigra*) sub-genomes (Fig. 1; Table 1). A recent study also reported the isolation of four functional homologues of a few glucosinolate pathway genes, including *GSL-ELONG* and *GSL-ALK*, which could be equally mapped into ‘A’- and ‘B’-genome-specific linkage groups (Bisht *et al.*, 2009). A high level of sequence conservation of the extant *BjuMYB28* was observed with their corresponding *MYB28* homologues from the *B. rapa* and *B. nigra* genomes, as confirmed by their close phylogenetic relationships (Fig. 2).

It is presumed that the divergence of the *MYB28* homologues in the *Brassica* lineage might have occurred during the genome triplication events that occurred in the ancestral *Brassica* species around ~13–17 MYA (Lysak *et al.*, 2005). As a consequence, the diploid *Brassica* species have retained 2–3 divergent copies (paralogues) of most of the genes in their genomes (Zang *et al.*, 2009; Wang *et al.*, 2011). The recent sequencing of *B. rapa* (http://Brassicadb.org/) reported three *MYB28*-like sequences (accession nos. Bra012961, Bra029311, and Bra035929), two of which could be successfully amplified in the current study. Only two MYB28 homologues from the *Brassica* C-genome (*B. oleracea*) are reported in publicly available databases. Using phylogeny, the orthologous MYB28 proteins pairs of the *Brassica* A-, B-, and C-genomes could be determined in this study. Considering the polyploidy level and complex genome architecture of *B. juncea*, the possibility of as yet unidentified *BjuMYB28* sequences that may exist as pseudogenes or functional genes cannot be completely ruled out. Nevertheless, our results provide substantial information on the *MYB28* homologues of *B. juncea*. With the advent of enriched genomic resources, a complete inventory of MYB28 homologues from various *Brassica* species will be possible in the near future, which could explain the variability of glucosinolate content across *Brassica* crops.

### *BjuMYB28* genes all control aliphatic glucosinolate accumulation

Polyploidy is an evolutionary process that plays a key role in generating the diversity of plant species (Adams and Wendel, 2005). The evolutionary consequences of duplicated genes after polyploidy include loss or silencing, maintaining ancestral function, and functional divergence. Over time, the function of structurally diverged homologues in allopolyploids can diverge from the ancestral gene, either through subfunctionalization or neo-functionalization (Lynch and Conery, 2000; Adams, 2007).

In the current study, over-expression of *BjuMYB28* homologues in two different genetic backgrounds of the phylogenetically close model system *A. thaliana,* demonstrated that the encoded BjuMYB28 proteins are involved in controlling aliphatic (Met-derived) glucosinolate biosynthesis in *B. juncea*, without directly affecting indolic glucosinolate biosynthesis. The *BjuMYB28* genes positively regulate the genes involved in the chain-elongation (*MAM1* and *MAM3*) and the formation of the core structure (*AtSt5b* and *AtST5c*) of aliphatic glucosinolates. The functional data in *A. thaliana* clearly showed that all four BjuMYB28 proteins are positive regulators of the genes involved in aliphatic glucosinolate biosynthesis, controlling the accumulation of both short- and long-chain aliphatic glucosinolates as also reported for the *Arabidopsis* homologue, AtMYB28 (Gigolashvili *et al.*, 2007*b*). Thus polyploidization of *Brassica* genomes has not altered basic *MYB28* gene function, and all homologues of the *MYB28* gene seem to retain subdivision of gene function in polyploid *Brassica* crops.

Amino acid sequence alignment of the four BjuMYB28 proteins showed significant structural variation in their C-terminal half. This variation might be responsible for their differential activation/regulatory control of aliphatic glucosinolate biosynthesis across plant development stages, tissue/cell types or during variable environmental conditions. Variable accumulation of total as well as individual aliphatic glucosinolates in the *Arabidopsis BjuMYB28* over-expression lines was observed in this study (Figs 4, 5). Further experiments in *B. juncea* will gain more insight about the functional divergence of A- and B-genome-specific *MYB28s*. Molecular characterization of more *MYB28*-like sequences from related *Brassica* species will help to understand the significance of variable C-terminal sequences for the regulation of aliphatic glucosinolate biosynthesis.

### Expression divergence of *MYB28* genes across diploid and allotetraploid *Brassica* species

Studies in allopolyploids have shown that homoeologous genes can be expressed at different levels and can respond differentially to polyploidy in various organs of the plant or in response to various environmental stimuli (Adams, 2007; Kliebenstein, 2008). Global-wide analysis of gene expression in cotton, wheat, and *B. napus* clearly shows that there are both immediate and long-term alterations in the expression of homoeologous genes arising from polyploidy, such as differential expression, transcriptional bias or gene silencing of homoeologues (Chaudhary *et al.*, 2009; Akhunova *et al.*, 2010; Higgins *et al.*, 2012). The majority of these alterations are known to be caused by *cis*-regulatory divergence between the diploid progenitors, thereby giving rise to transcriptional sub-functionalization.

**Fig. 9.**
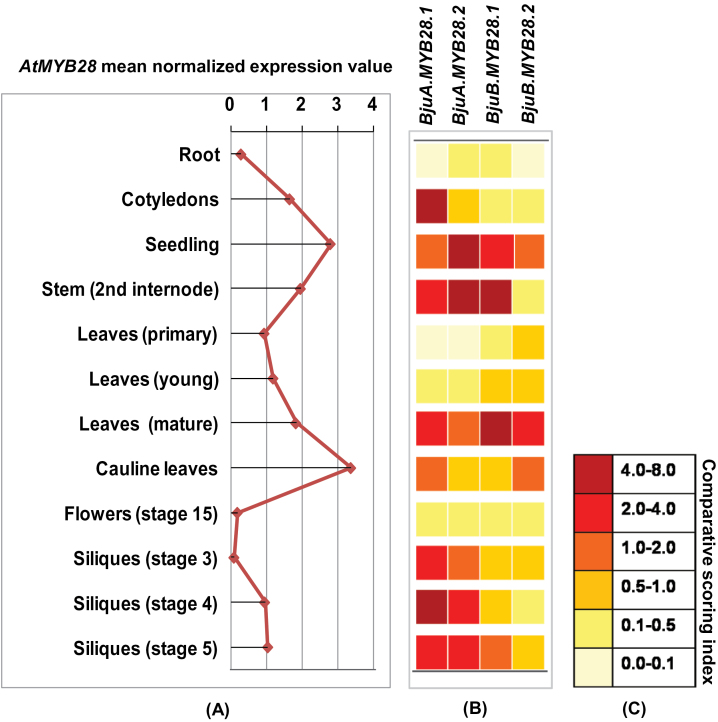
Graphical comparison of expression profiles of *BjuMYB28* genes across plant developmental stages in *B. juncea*. (A) The mean normalized expression value of *AtMYB28* (identifier 247549_at) were obtained by normalizing absolute expression values to median across different tissue types available in the AtGenExpress Visualization tool (www.arabidopsis.org/), and plotted. (B) Graphical representation of the expression profiles of the four *MYB28* homologues in *B. juncea* during the corresponding developmental stages. The colour of the box (data summarized from Figs 6 and 7) represents the comparative expression score of *BjuMYB28* genes. The two ‘A’ and ‘B’ subgenome specific homologues are also marked. (C) The comparative scoring index was constructed from the fold expression values of *BjuMYB28* genes obtained using real-time expression data as indicated.

Besides having variation in their coding regions, the gene structures of the *BjuMYB28*s have diverged in several other ways, including the promoter and intronic sequences. For example, when approximately 1kb of upstream sequence of the four *BjuMYB28* genes was scanned in the PLACE (PLant Cis-Acting Regulatory Elements) database (Higo *et al.*, 1991), several *cis*-regulatory elements related to tissue-dependent expression and elements responsive to glucose signalling, abiotic and biotic stress response, and sulphur assimilation were observed (see Supplementary Table S6 at *JXB* online). The disparity of various *cis*-regulatory elements observed among the four *BjuMYB28* promoters in all probability contribute to the differential expression patterns of *BjuMYB28*s as revealed by qRT-PCR analysis in *B. juncea* and GUS histochemical analysis of Pro_*BjuMYB28*_ *:GUS* transgenic lines developed in *A. thaliana* (Fig. 8). For example, the four *BjuMYB28* homologues have distinct expression patterns (Fig. 9), within leaves and other organs across development.

Glucosinolate formation in *B. juncea* leaves seems to be highly localized under the co-ordinated control of the *BjuMYB28* genes (Fig. 8B). Glucosinolate biosynthesis in the mid-vein of a leaf is largely associated with *BjuA.MYB28.2*, whereas all four *BjuMYB28* genes are associated with glucosinolate formation in the outer lamina of a leaf. The non-uniform distribution of glucosinolates in *A. thaliana* leaves has been reported earlier, wherein the major glucosinolates were found to be more abundant in tissues of the mid-vein and the outer lamina of the leaf than the inner lamina (Shroff *et al.*, 2008). This distribution has been at least partially attributed to the specific spatial expression of the *MYB*s that control aliphatic glucosinolate formation, including *AtMYB28*, *AtMYB29*, and *AtMYB76* (Sonderby *et al*., 2010*a*). The spatial expression patterns of *BjuMYB28* genes within the leaf also suggest a role in influencing the variable distribution of leaf glucosinolate content in *B. juncea* which, in turn, has important consequences for plant defence. When the feeding pattern of *Helicoverpa armigera* (the cotton bollworm) was studied on *A. thaliana*, the larvae avoided feeding on the mid-vein and periphery of the rosette leaves and fed mainly on the inner lamina. This feeding pattern was a direct consequence of the concentration and distribution of glucosinolates as determined using MALDI-TOF imaging (Shroff *et al.*, 2008).

Interestingly, the two A-genome *MYB28* homologues retained almost similar expression patterns post-polyploidization, whereas the B-genome homologues, particularly *BjuB.MYB28.1* have altered expression patterns in *B. juncea* compared with that in *B. nigra*. When the 5′ upstream region (1kb) of the *BjuMYB28* genes was compared across *B. juncea* and in species harbouring progenitor genomes, the upstream sequences of A-genome-specific homologues were found to be completely identical, whereas the B-genome-specific homologues showed a little divergence (see Supplementary Fig. S5 and Supplementary Table S5 at *JXB* online). In addition to the variable 5′ upstream regions, other factors like *trans*-regulatory elements and DNA methylation patterns could also explain the differential expression of homeologous gene pairs in allopolyploid genomes. Comparing the transcripts of the *BjuMYB28* genes from the two genomes, revealed that the both A-genome-specific transcripts in general are overrepresented across *B. juncea* development compared with B-genome transcripts (Fig. 9). This expression bias of A-subgenome-specific *MYB28* homologues potentially suggests its higher transcriptional contribution for controlling the aliphatic glucosinolate biosynthesis in allopolyploid *B. juncea*.

In addition to the specific cell- and tissue-level expression of A- and B-genome originating *BjuMYB28s*, all four genes showed higher expression levels with the onset of the reproductive phase. This trend may indicate a greater need for plant defence at the critical times of flowering and seed formation. Aliphatic glucosinolates are found throughout the plant, but the highest accumulation is found in the mature seeds (Brown *et al.*, 2003).

### *BjuMYB28*: a potential candidate for engineering low glucosinolate trait in *B. juncea*

Recent studies on the association of glucosinolate pathway genes with seed-glucosinolate QTLs in *B. juncea* and *B. napus* suggested that *MYB28*, particularly the A-genome-specific orthologues from these two complex allopolyploid genomes, are the major genetic determinants controlling glucosinolate variability (Ramchiary *et al.*, 2007; Bisht *et al.*, 2009; Feng *et al.*, 2012; Harper *at al*., 2012). In *B. juncea* out of the six QTLs identified for seed glucosinolate content, two QTLs namely *J3Gsl2* (A2) and *J17Gsl5* (B7) were found to contain the *MYB28* homologues (Ramchiary *et al.*, 2007; Bisht *et al.*, 2009). Recently, Harper *et al.* (2012) employing an associative transcriptomics approach to traits in *B. napus*, identified genomic deletions that underlie two quantitative trait loci for the glucosinolate content of seeds. Both the QTLs (occupying linkage groups A9 and C2) were found to contain *B. napus* orthologues of the transcription factor *MYB28* that had been lost from the low-glucosinolate accessions of *B. napus*. Based on previous reports and knowledge generated in this study, an in-depth characterization of *BjuMYB28* genes in native *B. juncea* is currently being performed. Our preliminary data suggested that the A-genome-specific *MYB28* homologues can be exploited for developing low glucosinolate lines in *B. juncea* (Augustine *et al.*, 2013).

The work described in this study has increased our understanding of BjuMYB28 regulatory mechanisms operational in the glucosinolate pathway in allopolyploid *B. juncea*. Our findings provide functional evidence of expression partitioning and subfunctionalization of *MYB28* gene family homologues in regulating aliphatic glucosinolate content in *B. juncea*. The information obtained in the current study should facilitate tissue-specific engineering of aliphatic glucosinolate traits in *Brassica* crops using conventional breeding and/or transgenic approaches in order to reduce anti-nutritive seed glucosinolates to economically acceptable levels while insuring that defence of leaves and other tissues are not compromised.

## Supplementary data

Supplementary data can be found at *JXB* online.

Supplementary Table S1. List of primers used in the current study.

Supplementary Table S2. Nucleotide sequence identity (%) of coding DNA sequences (CDS) of *MYB28* homologues isolated from *B. juncea* (Bju), *B. rapa* (Bra), and *B. nigra* (Bni).

Supplementary Table S3. Nucleotide sequence identity (%) of *BjuMYB28* full-length genes.

Supplementary Table S4. Primer amplification efficiency test of *BjuMYB28* genes used in the current study.

Supplementary Table S5. Nucleotide sequence identity (%) of the 5′ upstream region of the *MYB28* homologues isolated from *B. juncea* and its progenitor genomes.

Supplementary Table S6. Summary of various *cis*-regulatory elements present within a 1kb upstream region of the *BjuMYB28* genes, obtained using the PLACE database (www.dna.affrc.go.jp/PLACE/).

Supplementary Fig. S1. Nucleotide sequence alignment of coding DNA sequences (CDS) of *MYB28* homologues isolated from *Brassica* species. The sequence alignment of CDS of *A. thaliana AtMYB28* (At5g61420), *B. juncea BjuMYB28(1–4*), *B. nigra BniMYB28(1,2*), and *B. rapa BraMYB28(1,2*) was performed using the ClustalW algorithm available in the MegAlign module of DNASTAR software (Lasergene). Dark shading represents conserved residues.

Supplementary Fig. S2. Nucleotide sequence alignment of full-length genomic sequences of four *BjuMYB28* genes. The sequence alignment of *AtMYB28* (At5g61420), and full-length *B. juncea BjuMYB28* genes was performed using the ClustalW algorithm available in the MegAlign module of DNASTAR software (Lasergene). The positions of two introns are marked within the brackets []. Nucleotide in a dark background represents residues differing from the consensus.

Supplementary Fig. S3. Indolic glucosinolate profiles of *BjuMYB28* over-expression (OE) lines in an *Arabidopsis* wt (Col-0) background. Two independent transgenic events for each BjuMYB28 homologue were analysed and the value represent mean ±SE (*n* ≥4).

Supplementary Fig. S4. Transcript levels of glucosinolate pathway genes in rosette leaves of representative (A) *BjuMYB28* mutant complementation (MC) and (B) over-expression (OE) lines in *A. thaliana*. qRT-PCR analysis of aliphatic glucosinolate pathway genes was performed and the transcript accumulation was measured with reference to the *Arabidopsis* wild-type Col-0 and BRC_H161b mutant background, respectively (both set at 1). Values are mean ±SE of three independent biological replicates. Asterisks indicate significant differences in gene expression compared with the respective background (*P* <0.05, in Fishers LSD test).

Supplementary Fig. S5. Nucleotide sequence alignment of the 5′ upstream region of four *MYB28* homologues from *B. juncea* and its progenitor genomes. The sequence alignment was performed using the ClustalW algorithm available in the MegAlign module of DNASTAR software (Lasergene). Nucleotide in a dark background represents residues differing from the consensus.

Supplementary Data
