# Supplementary material for: Four genes encoding MYB28, a major transcriptional regulator of the aliphatic glucosinolate pathway, are differentially expressed in the allopolyploid Brassica juncea
Source: J Exp Bot. 2013 Sep 16;64(16):4907–21. doi: 10.1093/jxb/ert280 (PMC3830477; doi:10.1093/jxb/ert280)
Supplement: Supplementary Data [file supp_64_16_4907__index.html]

Four genes encoding MYB28, a major transcriptional regulator of the aliphatic glucosinolate pathway, are differentially expressed in the allopolyploid Brassica juncea — Four genes encoding MYB28, a major transcriptional regulator of the aliphatic glucosinolate pathway, are differentially expressed in the allopolyploid Brassica juncea — Supplementary Data 

# Four genes encoding MYB28, a major transcriptional regulator of the aliphatic glucosinolate pathway, are differentially expressed in the allopolyploid *Brassica juncea*

## Supplementary Data

Data files

**Files in this Data Supplement:**

- Supplementary Data - Supplementary Data
