# Supplementary material for: Four genes encoding MYB28, a major transcriptional regulator of the aliphatic glucosinolate pathway, are differentially expressed in the allopolyploid Brassica juncea
Source: J Exp Bot. 2013 Sep 16;64(16):4907–21. doi: 10.1093/jxb/ert280 (PMC3830477; doi:10.1093/jxb/ert280)
Supplement: Supplementary Data [file supp_ert280_jexbot100404_file001.pdf]

Four genes encoding MYB28, a major transcriptional regulator of aliphatic glucosinolate pathway, are differentially expressed in the allopolyploid *Brassica juncea*

Rehna Augustine, Manoj Majee, Jonathan Gershenzon and Naveen C. Bisht

SUPPLEMENTAL DATA

Table S1. List of primers used in the current study.

Table S2: Nucleotide sequence identity (%) of coding DNA sequences (CDS) of *MYB28* homologs isolated from *B. juncea* (Bju), *B. rapa* (Bra) and *B. nigra* (Bni).

Table S3: Nucleotide sequence identity (%) of *BjuMYB28* full-length genes.

Table S4. Primer amplification efficiency test of *BjuMYB28* genes used in the current study.

Table S5: Nucleotide sequence identity (%) of 5' upstream region of *MYB28* homologs isolated from *B. juncea* and its progenitor genomes.

Table S6. Summary of various *cis*-regulatory elements present within 1 kb upstream region of *BjuMYB28* genes, obtained using PLACE database (www.dna.affrc.go.jp/PLACE/).

Figure S1. Nucleotide sequence alignment of coding DNA sequences (CDS) of *MYB28* homologs isolated from *Brassica* species. The sequence alignment of CDS of *A. thaliana AtMYB28* (At5g61420), *B. juncea BjuMYB28(1-4*), *B. nigra BniMYB28(1,2*) and *B. rapa BraMYB28(1,2*) was performed using the ClustalW algorithm available in MegAlign module of DNASTAR software (Lasergene). Dark shade represents conserved residues.

Figure S2. Nucleotide sequence alignment of full-length genomic sequences of four *BjuMYB28* genes. The sequence alignment of *AtMYB28* (At5g61420), and full length *B. juncea BjuMYB28* genes was performed using the ClustalW algorithm available in MegAlign module of DNASTAR software (Lasergene). The positions of two introns are marked within

the brackets [ ]. Nucleotide in dark background represents residues differing from the consensus.

Figure S3. Indolic glucosinolate profiles of *BjuMYB28* overexpression (OE) lines in *Arabidopsis* wt (Col-0) background. Two independent transgenic events for each BjuMYB28 homolog was analyzed and the value represent mean $\pm$ SE ($n \geq 4$).

Figure S4. Transcript levels of glucosinolate pathway genes in rosette leaves of representative (A) *BjuMYB28* mutant complementation (MC) and (B) over- expression (OE) lines in *A. thaliana*. qRT-PCR analysis of aliphatic glucosinolate pathway genes was performed and the transcript accumulation was measured with reference to the *Arabidopsis* wild-type Col-0 and BRC_H161b mutant background, respectively (both set at 1). Values are mean ± SE of three independent biological replicates. Asterisks indicate significant differences in gene expression compared to the respective background ($P < 0.05$, in Fishers LSD test).

Figure S5. Nucleotide sequence alignment of 5' upstream region of four *MYB28* homologs from *B. juncea* and its progenitor genomes. The sequence alignment was performed using the ClustalW algorithm available in MegAlign module of DNASTAR software (Lasergene). Nucleotide in dark background represents residues differing from the consensus.

Table S1: List of primers used in the current study.

S. No.	Primer code	Sequence 5'-3'
1	Myb28 FP	GAAGAAAGGGGCATGGACC
2	Myb28 RP	GATCCATACCGAGATCAGAAGTAG
3	AtMyb28 F	CACCATGTCAAGAAAGCCATGTTGCGTC
4	AtMyb28 RNS	TATGAAATGCTTTTCAAGCGA
5	Myb28-1/3 YFP FP	CACCATGTCAAGAAAGCCATGT
6	Myb28-2/4 YFP FP	CACCATGTCAAGAAAACCGTGT
7	Myb 28-1 YFP RP	TATGATTTGCTTCTCCAGGAAATC
8	Myb28-2 YFP RP	TATGAGATGCTTTTCGAAGGAATC
9	Myb28-3 YFP RP	TATGATTTGCTTATCGAAGAAATC
10	Myb28-4 YFP RP	TATGAGGGAATCAGACTCCGTGTC
11	Myb28-1/2 CDS FP	ATGTCAAGAAAACCGTGTTGTGTCG
12	Myb 28-1 CDS RP	TCATATGATTTGCTTCTCCAGGA
13	Myb 28-2 CDS RP	GCATGAAGATTCATATGAGATGCTT
14	Myb28-3 CDS FP	ATGTCAAGAAAGCCATGTTGTGTCG
15	Myb 28-3 CDS RP	GGATCCTCATATGATTTGCTTATCGAAG
16	Myb28-4 CDS FP	ATTACTCGAGATGTCAAGAAAACCGTG
17	Myb 28-4 CDS RP	ATGCTCTAGATCATATGAGGGAATCAG
18	Myb 28-15'GWP1	CCTTTTTAACCCTAATTAAACACCATATTCTC
19	Myb 28-15'GWP2	AAATTATATACTAACCAGATTTTTGGGGC
20	Myb 28-25'GWP1	CCCACACCTTTTCAGCCCTAGTAAAAC
21	Myb 28-25'GWP2	ATTCAACATATGATTTTCATACATCAAATAC
22	Myb 28-35'GWP1	TTTCAACCCTAATTAAACACCATATTCG
23	Myb 28-35'GWP2	ACCATATTCGCATCAAAATATTATACAATAC
24	Myb 28-45'GWP1	TCCACACCTTTTTAACCCTAATTACAT
25	Myb 28-45'GWP2	AGTACGATTACTAAATAAGCATGTGTATGA
26	Myb 28-25'GWP1 (2)	GGTACTAGAAGAAACACTTAAATCACC
27	Myb 28-25'GWP2 (2)	CATACGACAAAACAAATTGGAGTTGGTTCGG
28	Myb 28-35'GWP1 (2)	GATGGTGGTGAGCTTAAGAGCTATTCTCT
29	Myb 28-35'GWP2 (2)	CAAGAAAAATATTTGCAAATTGATGCTGT
30	Myb 28-45'GWP1 (2)	ATTAGGGATGGCGAAAGAAATCGA
31	Myb 28-45'GWP2 (2)	GTAAATCTAACCGGAGTGAAATCGAT
32	Myb28-1 RACE P1	ACCTGCGATGGACTAACTACCTAAAACCTG
33	Myb28-1 RACE P2	ACTTCAACACAGTTTCCGAGATTACAAGG
34	Myb28-2 RACE P1	TGCAAGAAACGTTTTAAGAAATCCAG
35	Myb28-2 RACE P2	AAGGTACCATGAGTGCTAATACAAGCTTTTCC
36	Myb28-2 RACE P3	TGAGGAGCACAACATGAATCATGAGTATG
37	Myb28-3 RACE P1	CGGTTTCAACCCGGCTTCCGAGATCAC
38	Myb28-3 RACE P2	GCAAGAAAAGTATTAAGAAATCGAACTC
39	Myb28-4 RACE P1	TGCTTCTCGTGGTAACAAGTGGTCGGA
40	Myb28-4 RACE P2	CCAACCATACTGTCAACACGCCTCC
41	Myb28-4 RACE P3	ACAAAGATGGGACACCAGTTCAAGGCG

42	Myb28-1UTR FP	ATCTATATATTCGAACAAACGGT
43	Myb28-1UTR RP	CCTATTAACCCTTATTTAACAT
44	Myb28-2EX FP	AAATCCTACTGTCACCACGTCG
45	Myb28-3EX RP	ATTGCTGGTAGTCTCGGAAAC
46	Myb28-3UTR FP	ATCTTTTTATATTCGAACTG
47	Myb28-3UTR RP	ACATGAGTTCTCGCCTTCT
48	Myb28-4UTR FP	GAGTCTTCATTACCAAACAG
49	Myb28-4UTR RP	TAAACTCGGGATTACTAAC
50	Bjactin FP	CTTCTTACCGAGGCTCCTCT
51	Bjactin RP	AAGGATCTTCATGAGGTAATCAGT
52	Myb28-1Pt GUS FP	ACCATGGATCAAGGTGGGTTCCAAAC
53	Myb28-1Pt GUS RP	ACTGCAGTTTCTCCGATATATGTGAAC
54	Myb28-2Pt GUS FP	CCATGGACAAGAGTGGCAAACAAATAG
55	Myb28-2Pt GUS RP	ACTGCAGTATTTCCGATATATATGAAC
56	Myb28-3Pt GUS FP	ACCCGGGTGTGGAAGATGCAGTATA
57	Myb28-3Pt GUS RP	ACTCGAGGCTTTCTTGACATTTTCTCCGA
58	Myb28-4Pt GUS FP	ACCCGGGCTATGCGGTTAGAGAACTATGA
59	Myb28-4Pt GUS RP	ACTCGAGTCGCTATATATGCACACTAGTAGGCA
60	AtMAM1FP	CGGCTGAAAGAGTGGGGATATGA
61	AtMAM1RP	CGTTAGCGCCGTTTAATTTCTC
62	AtCYP79F1FP	CCATACCCTTTTCACATCCTACTAGTCT
63	AtCYP79F1RP	GTAGATTGCCGAGGATGGGC
64	AtCYP83A1FP	TTCAAGAGGTTGTCAATGAGACGC
65	AtCYP83A1RP	CTACAATATCCAAGATGACGGCTTT
66	AtMAM3 FP	GAGAAATTGAACGCTGTCTTCTCAC
67	AtMAM3 RP	AGCCGTTAGACTTTAAACCGTTAGC
68	AtCYP79F2 FP	ACTAGGATTTATCGTCTTCATCGCA
69	AtCYP79F2 RP	CTAGGACGAGTCATGATTAGTTCGG
70	AtST5b FP	GGAATCCAAAACCATAAACGACG
71	AtST5b RP	CGGATCTTTTGGTCTCCAGCC
72	AtST5c FP	CCCTACCGAGTCACGACGAGA
73	AtST5c RP	GGTAGCCACCAGTAACCACCATACT
74	AtActin2FP	TAACTCTCCCGCTATGTATGTCGC
75	AtActin2RP	CCACTGAGCACAATGTTACCGTAC

Table S2: Nucleotide sequence identity (%) of coding DNA sequences (CDS) of four *MYB28* homologs isolated from *B. juncea* (Bju), *B. rapa* (Bra) and *B. nigra* (Bni). The sequence alignment was done using the ClustalW module of DNASTAR software (Lasergene). Value in shaded box represents % identity between MYB28 homologs from diploid progenitor and allotetraploid *B. juncea*.

	At5g61420	***BjuMYB28-1***	***BjuMYB28-2***	***BjuMYB28-3***	***BjuMYB28-4***	***BniMYB28-1***	***BniMYB28-2***	***BraMYB28-1***	***BraMYB28-2***
At5g61420 (AtMYB28)	***	77.4	85.6	80.0	82.5	77.5	85.7	80.1	82.4
BjuMYB28-1 (BjuB.MYB28.1)		***	83.9	89.5	79.8	**99.9**	84.0	89.6	79.7
BjuMYB28-2 (BjuB.MYB28.2)			***	83.5	84.3	80.7	**99.7**	83.6	84.2
BjuMYB28-3 (BjuA.MYB28.1)				***	81.2	88.5	86.1	**99.9**	81.1
BjuMYB28-4 (BjuA.MYB28.2)					***	79.0	86.9	81.3	**99.9**
BniMYB28-1 (BniB.MYB28.1)						***	84.1	89.6	79.8
BniMYB28-2 (BniB.MYB28.2)							***	83.8	84.4
BraMYB28-1 (BraA.MYB28.1)								***	81.2
BraMYB28-2 (BraA.MYB28.2)									***

Table S3: Nucleotide sequence identity (%) of *BjuMYB28* full-length genes. The sequence alignment was done using the ClustalW module of DNASTAR software (Lasergene).

	AtMYB28	***BjuMYB28-1***	***BjuMYB28-2***	***BjuMYB28-3***	***BjuMYB28-4***
AtMYB28	***	73.4	79.1	73.8	74.4
BjuMYB28-1 (BjuB.MYB28.1)		***	63.4	79	65.8
BjuMYB28-2 (BjuB.MYB28.2)			***	78.8	79.1
BjuMYB28-3 (BjuA.MYB28.1)				***	69.8
BjuMYB28-4 (BjuA.MYB28.2)					***

Table S4: Primer amplification efficiency test of *BjuMYB28* gene homologs used in the current study. A known amount of template (purified plasmid DNA) and *BjuMYB28* homolog specific primers were used. A 1:50 diluted cDNA of seedling was used to quantify the transcript levels of *BjuMYB28* homologs.

**Template amount**	**BjuA.MYB28.1**	**BjuA.MYB28.2**	**BjuB.MYB28.1**	**BjuB.MYB28.2**
**100pg**	10.344	10.522	10.718	12.559
**10pg**	14.352	13.654	14.396	15.93
**1pg**	17.955	17.836	18.62	19.3
**100fg**	22.049	22.096	23.227	22.661
**10fg**	25.568	24.925	26.095	25.917
**1fg**	27.931	28.72	29.893	29
R2	0.9945	0.9972	0.9968	0.9997
Slope(y)	3.49x+7.13	3.68x+6.72	3.87x+6.93	3.30x+9.34
**seedling**	26.187	26.657	27.5	26.791

Table S5: Nucleotide sequence identity (%) of 5' upstream region of *MYB28* homologs isolated from *B. juncea* and its progenitor genomes. The sequence alignment was done using the ClustalW module of DNASTAR software (Lasergene).

	ptrBjuB.MYB28.1	ptrBniB.MYB28.1	ptrBjuB.MYB28.2	ptrBniB.MYB28.2	ptrBjuA.MYB28.1	ptrBraA.MYB28.1	ptrBjuA.MYB28.2	ptrBraA.MYB28.2
**ptrBjuB.MYB28.1**	***	99.7	55.2	54.6	65.5	65.6	50.9	51.0
**ptrBniB.MYB28.1**		***	55.1	54.7	65.4	65.5	50.7	50.8
**ptrBjuB.MYB28.2**			***	98.8	54.9	54.9	59.6	59.5
**ptrBniB.MYB28.2**				***	55.7	55.7	60.4	60.5
**ptrBjuA.MYB28.1**					***	99.8	51.9	51.9
**ptrBraA.MYB28.1**						***	51.9	51.9
**ptrBjuA.MYB28.2**							***	99.7
**ptrBraA.MYB28.2**								***

Table S6: Summary of various cis-regulatory elements present within 1 kb upstream region of *BjuMYB28* homologs, obtained using PLACE database (www.dna.affrc.go.jp/PLACE/).

cis-elements	BjuA.MYB28.1	BjuA.MYB28.2	BjuB.MYB28.1	BjuB.MYB28.2
-10PEHVPSBD	3	1	2	1
-300ELEMENT		1	1	1
AACACOREOSGLUB1			2	
ABRELATERD1	1		2	2
ABREMOTIFAOSOSEM				1
ACGTABREMOTIFA2OSEM				2
ACGTATERD1	2		6	8
ACGTOSGLUB1	1			1
AGMOTIFNTMYB2				1
AMYBOX1	1		1	
AMYBOX2		1	1	
ANAERO1CONSENSUS	1		2	2
ANAERO2CONSENSUS	1	1	1	
ARR1AT	8	8	8	8
ASF1MOTIFCAMV			1	
BIHD1OS	1	1		2
BOXCPSAS1			1	
BOXIINTPATPB	2	2	1	2
BS1EGCCR			1	
CAATBOX1	16	13	14	12
CACTFTPPCA1	18	14	18	17
CAREOSREP1				3
CARGCW8GAT	2	2	2	8
CBFHV	1			
CCA1ATLHCB1		1		
CCAATBOX1	2	1		3
CGACGOSAMY3			1	3
CIACADIANLELHC	1			1
CTRMCAMV35S		1		
CURECORECR	6			6
DOFCOREZM	9	21	14	16
DPBFCOREDCDC3	1	1		
E2F1OSPCNA			1	
E2FANTRNR			1	
E2FCONSENSUS			1	
EBOXBNNAPA	4	6	12	8
EECCRCAH1	1	1		
ELRECOREPCRP1				1

ERELEE4	2			
EVENINGAT	1	1	1	
GADOWNAT				1
GARE1OSREP1	1	1		
GAREAT	1		2	2
GATABOX	12	12	15	11
GT1CONSENSUS	11	16	12	12
GT1CORE				1
GT1GMSCAM4	3	6	3	3
GT1MOTIFPSRBCS	1			
GTGANTG10	5	7	7	12
HDZIP2ATATHB2	1			1
HEXMOTIFTAH3H4			1	1
IBOX		2	1	2
IBOXCORE	1	4	2	5
IBOXCORENT		1		1
INRNTPSADB	1	3	4	3
L1BOXATPDF1			1	1
LECPLEACS2	1	1	1	
LTRE1HVBLT49			1	
LTRECOREATCOR15		1		1
MARTBOX	3	1		3
MYB1AT	2	1		4
MYB2CONSENSUSAT		1	1	
MYBATRD22				1
MYBCORE	1	3	2	1
MYBGAHV			1	
MYBPLANT				2
MYBPZM		1	1	
MYBST1	1	3	4	1
MYCCONSENSUSAT	4	6	12	8
NODCON1GM	1	2		
NODCON2GM	4	3	5	4
NTBBF1ARROLB		1	1	1
OSE1ROOTNODULE	1	2		
OSE2ROOTNODULE	4	3	5	4
P1BS			2	4
POLASIG1	1	2	2	6
POLASIG2	4			
POLASIG3	7	5	4	5
POLLEN1LELAT52	8	11	10	13
PREATPRODH	1			
PRECONSCRHSP70A		1		2

PYRIMIDINEBOXHVEPB1		1	1	
PYRIMIDINEBOXOSRAMY1A	2	2	3	
RAV1AAT	1	3	6	
RBCSCONSENSUS		2		
REALPHALGLHCB21	1	4		4
RHERPATEXPA7		1	1	1
ROOTMOTIFTAPOX1	21	12	13	13
RYREPEATBNNAPA		2	1	
RYREPEATGMGY2		1	1	
RYREPEATLEGUMINBOX		1	1	
S1FBOXSORPS1L21		1		
SEF1MOTIF	2			
SEF3MOTIFGM		1	1	1
SEF4MOTIFGM7S	3	4		3
SORLIP1AT		2	1	1
SORLIP2AT				1
SORLREP3AT	2		2	
SP8BFIBSP8AIB	2		1	
SPHCOREZMC1			1	
SREATMSD		1		
SURECOREATSULTR11	1	1	2	1
SV40COREENHAN				1
TAAAGSTKST1	1	4	2	6
TATABOX3	6		2	
TATABOX4	1	3		2
TATABOX5	8	2	5	8
TATABOXOSPAL	2		3	1
TATAPVTRNALEU		1		
TATCCACHVAL21		1		
TATCCAOSAMY		2	1	
TATCCAYMOTIFOSRAMY3D		1	1	
TBOXATGAPB	1	1	1	2
TGACGTVMAMY			1	
WBOXATNPR1				1
WBOXATNPR1			1	1
WBOXHVISO1				2
WBOXNTCHN48				1
WBOXNTERF3	1		1	3
WRKY71OS			2	5

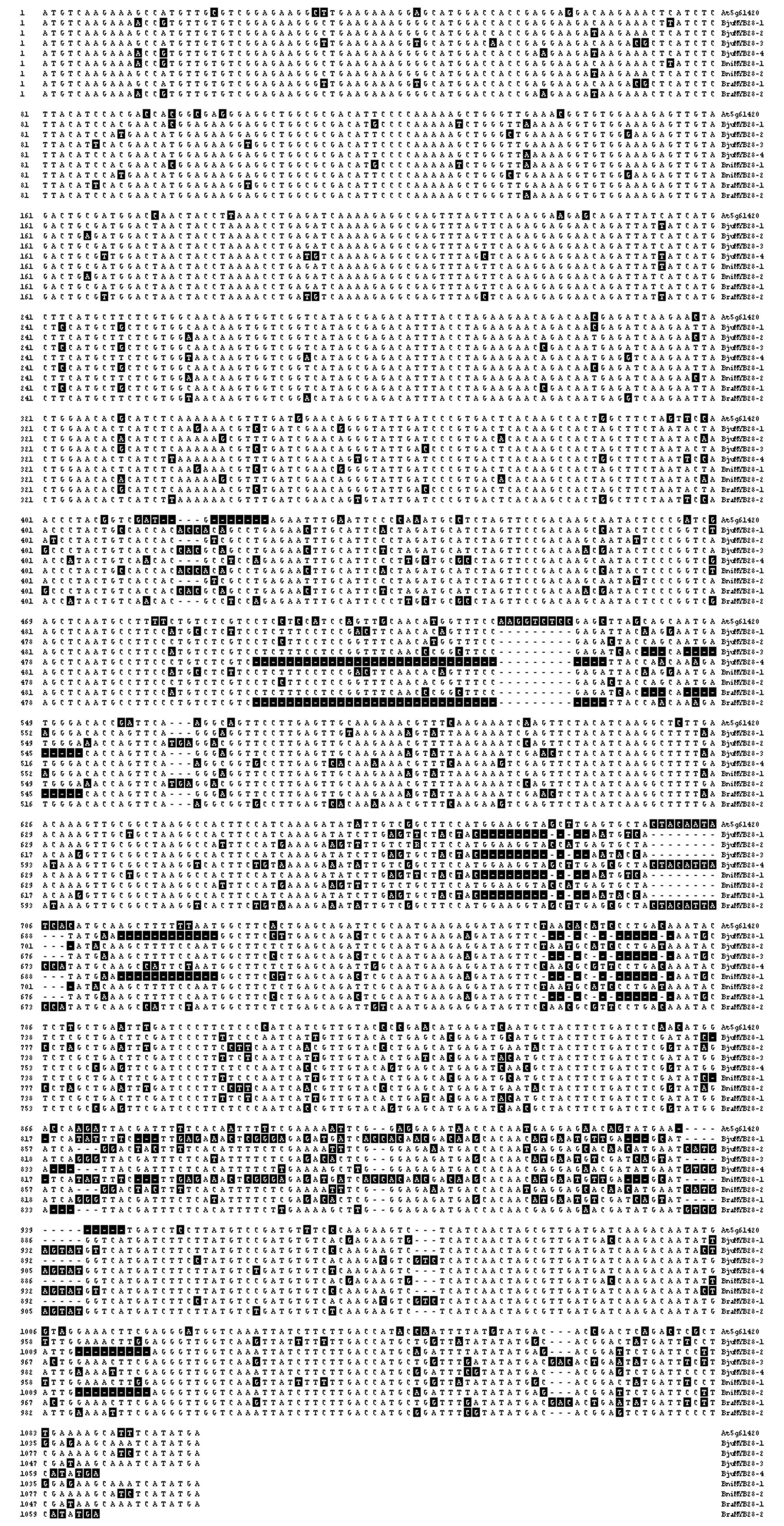
At5g61420
BjuMYB28-1
BjuMYB28-2
BjuMYB28-3
BjuMYB28-4
BniMYB28-1
BniMYB28-2
BraMYB28-1
BraMYB28-2

Figure S1

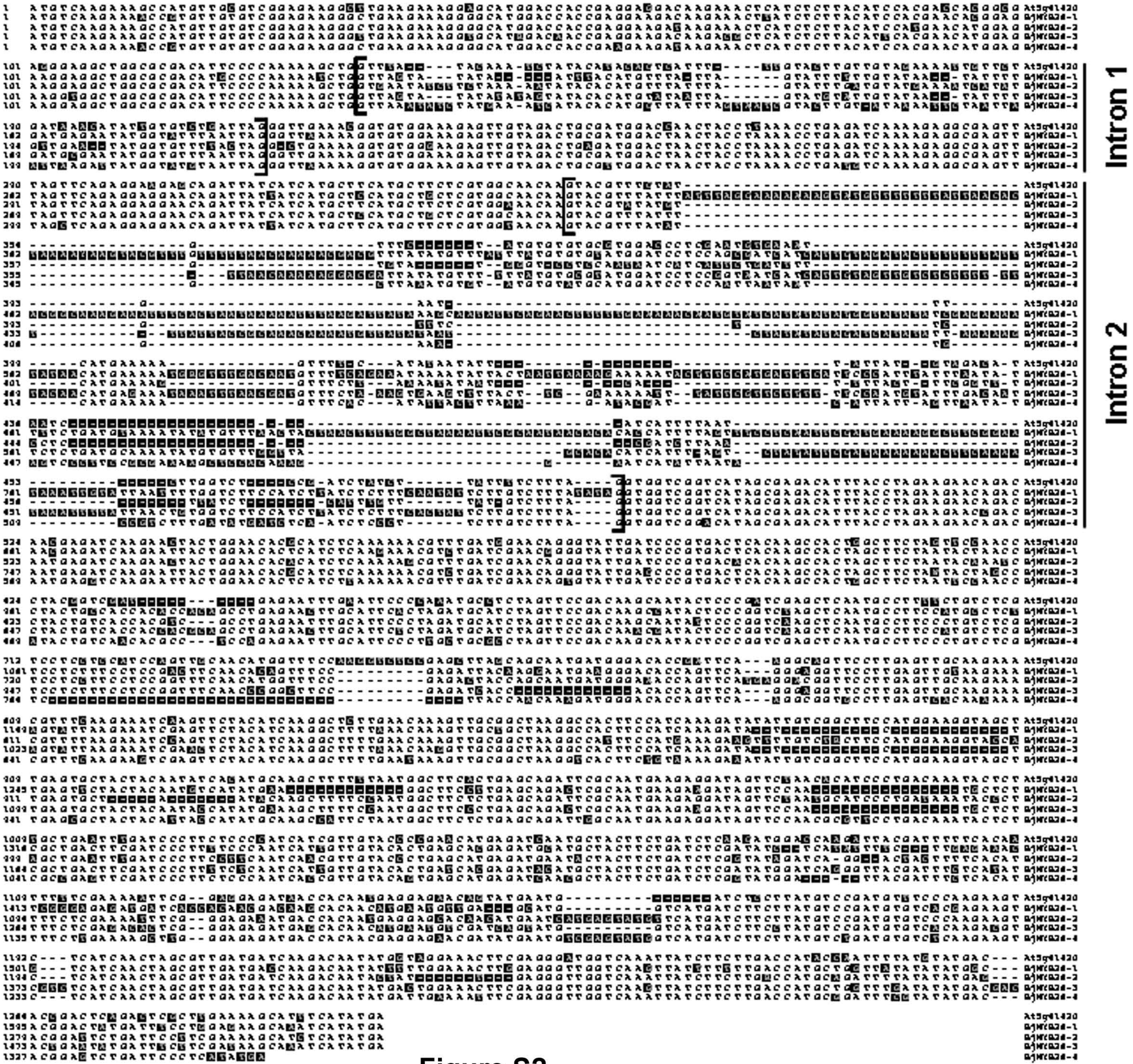

Figure S2

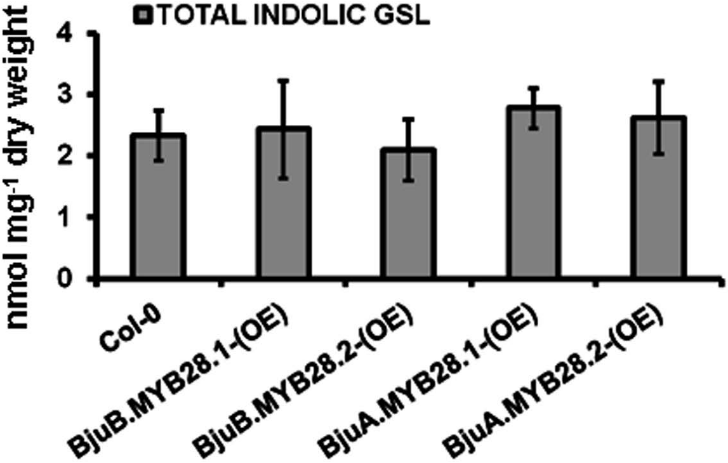
TOTAL INDOLIC GSL
nmol mg-1 dry weight
4
3
2
1
0
Col-0
BjuB.MYB28.1-(OE)
BjuB.MYB28.2-(OE)
BjuA.MYB28.1-(OE)
BjuA.MYB28.2-(OE)

Figure S3

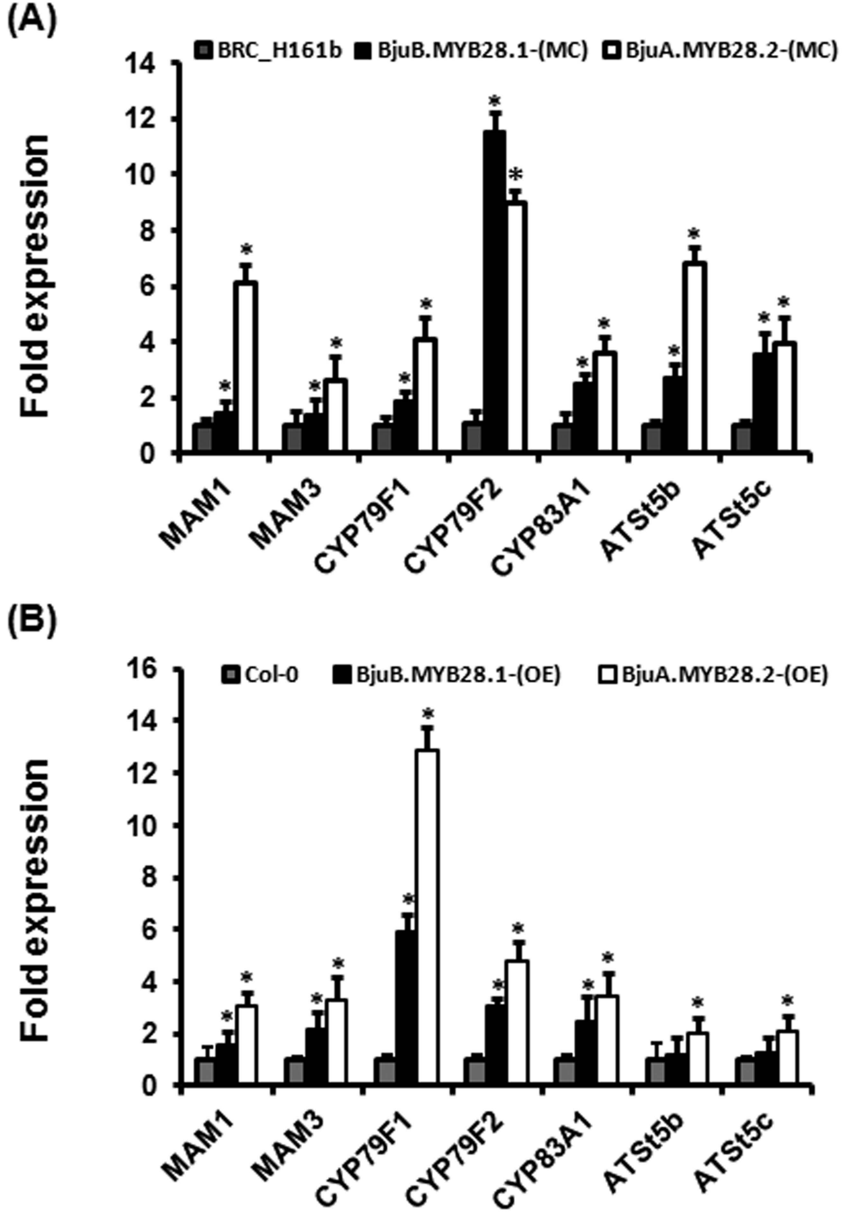
(A)
BRC_H161b
BjuB.MYB28.1-(MC)
BjuA.MYB28.2-(MC)
Fold expression
0
2
4
6
8
10
12
14
MAM1
MAM3
CYP79F1
CYP79F2
CYP83A1
ATSt5b
ATSt5c
(B)
Col-0
BjuB.MYB28.1-(OE)
BjuA.MYB28.2-(OE)
Fold expression
0
2
4
6
8
10
12
14
16
MAM1
MAM3
CYP79F1
CYP79F2
CYP83A1
ATSt5b
ATSt5c

Figure S4

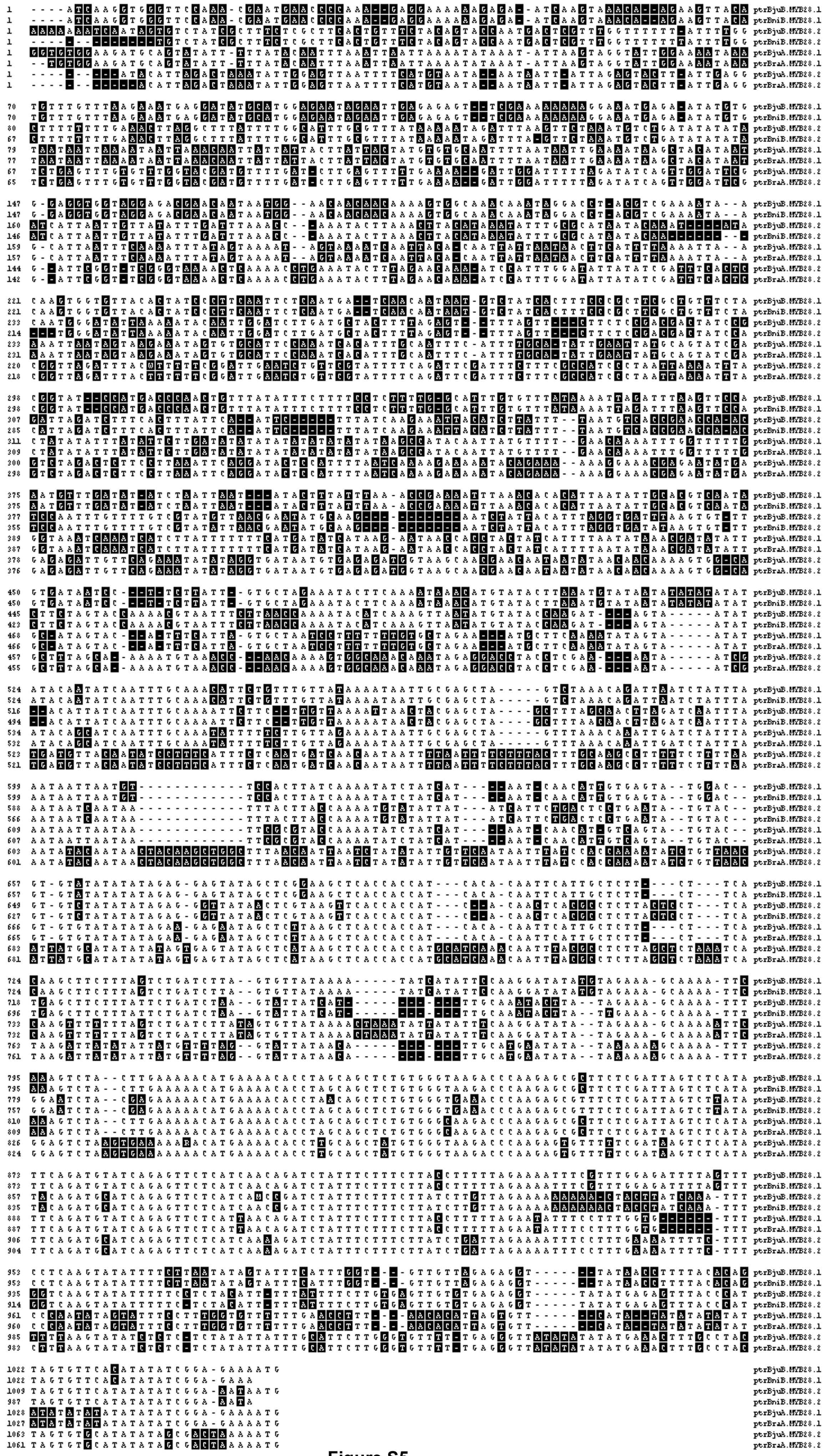

Figure S5
